# The direct binding of bioactive peptide Andersonin-W1 to TLR4 expedites the healing of diabetic skin wounds

**DOI:** 10.1186/s11658-024-00542-4

**Published:** 2024-02-05

**Authors:** Chao Li, Yuxin Xiong, Zhe Fu, Yuxin Ji, Jiayi Yan, Yan Kong, Ying Peng, Zeqiong Ru, Yubing Huang, Yilin Li, Ying Yang, Li He, Jing Tang, Ying Wang, Xinwang Yang

**Affiliations:** 1 Department of Anatomy and Histology and Embryology, Faculty of Basic Medical Science, Kunming Medical University, Kunming, 650500 Yunnan China; 2 Key Laboratory of Chemistry in Ethnic Medicinal Resources and Key Laboratory of Natural Products Synthetic Biology of Ethnic Medicinal Endophytes, State Ethnic Affairs Commission and Ministry of Education, School of Ethnic Medicine, Yunnan Minzu University, Kunming, 650504 Yunnan China; 3 Department of Biochemistry and Molecular Biology, Faculty of Basic Medical Science, Kunming Medical University, Kunming, 650500 Yunnan China; 4 Department of Endocrinology, Affiliated Hospital of Yunnan University, Kunming, 650021 Yunnan China; 5 Department of Dermatology, First Affiliated Hospital of Kunming Medical University, Kunming, 650032 Yunnan China

**Keywords:** Diabetic skin wound healing, TLR4/NF-κB, Inflammation, Angiogenesis

## Abstract

**Background:**

Chronic nonhealing wounds remain a considerable challenge in clinical treatment due to excessive inflammation and impeded reepithelialization and angiogenesis. Therefore, the discovery of novel prohealing agents for chronic skin wounds are urgent and important. Amphibian-derived prohealing peptides, especially immunomodulatory peptides, provide a promising strategy for the treatment of chronic skin trauma. However, the mechanism of immunomodulatory peptides accelerating the skin wound healing remains poorly understood.

**Methods:**

The prohealing ability of peptide Andersonin-W1 (AW1) was assessed by cell scratch, cell proliferation, transwell, and tube formation. Next, full-thickness, deep second-degree burns and diabetic full-thickness skin wounds in mice were performed to detect the therapeutic effects of AW1. Moreover, the tissue regeneration and expression of inflammatory cytokines were evaluated by hematoxylin and eosin (H&E), enzyme-linked immunosorbent assay (ELISA), and immunohistochemistry staining. Molecular docking, colocalization, and western blotting were used to explore the mechanism of AW1 in promoting wound healing.

**Results:**

We provide solid evidence to display excellent prohealing effects of AW1, identified as a short antimicrobial peptide in our previous report. At relative low concentration of nM, AW1 promoted the proliferation, migration, and scratch repair of keratinocyte, macrophage proliferation, and tube formation of HUVEC. AW1 also facilitated reepithelialization, granulation regeneration, and angiogenesis, thus significantly boosting the healing of full-thickness, deep second-degree burns and diabetic skin wounds in mice. Mechanistically, in macrophages, AW1 directly bound to Toll-like receptor 4 (TLR4) in the extracellular region and regulated the downstream nuclear factor‐κB (NF-κB) signaling pathway to facilitate the inflammatory factor secretion and suppress excessive inflammation induced by lipopolysaccharide (LPS). Moreover, AW1 regulated macrophage polarization to promote the transition from the inflammatory to the proliferative phase and then facilitated reepithelialization, granulation regeneration, and angiogenesis, thus exhibiting excellent therapeutic effects on diabetic skin wounds.

**Conclusions:**

AW1 modulates inflammation and the wound healing process by the TLR4/NF-κB molecular axis, thus facilitating reepithelialization, granulation regeneration, and angiogenesis. These findings not only provided a promising multifunctional prohealing drug candidate for chronic nonhealing skin wounds but also highlighted the unique roles of “small” peptides in the elucidation of “big” human disease mechanisms.

**Graphical Abstract:**

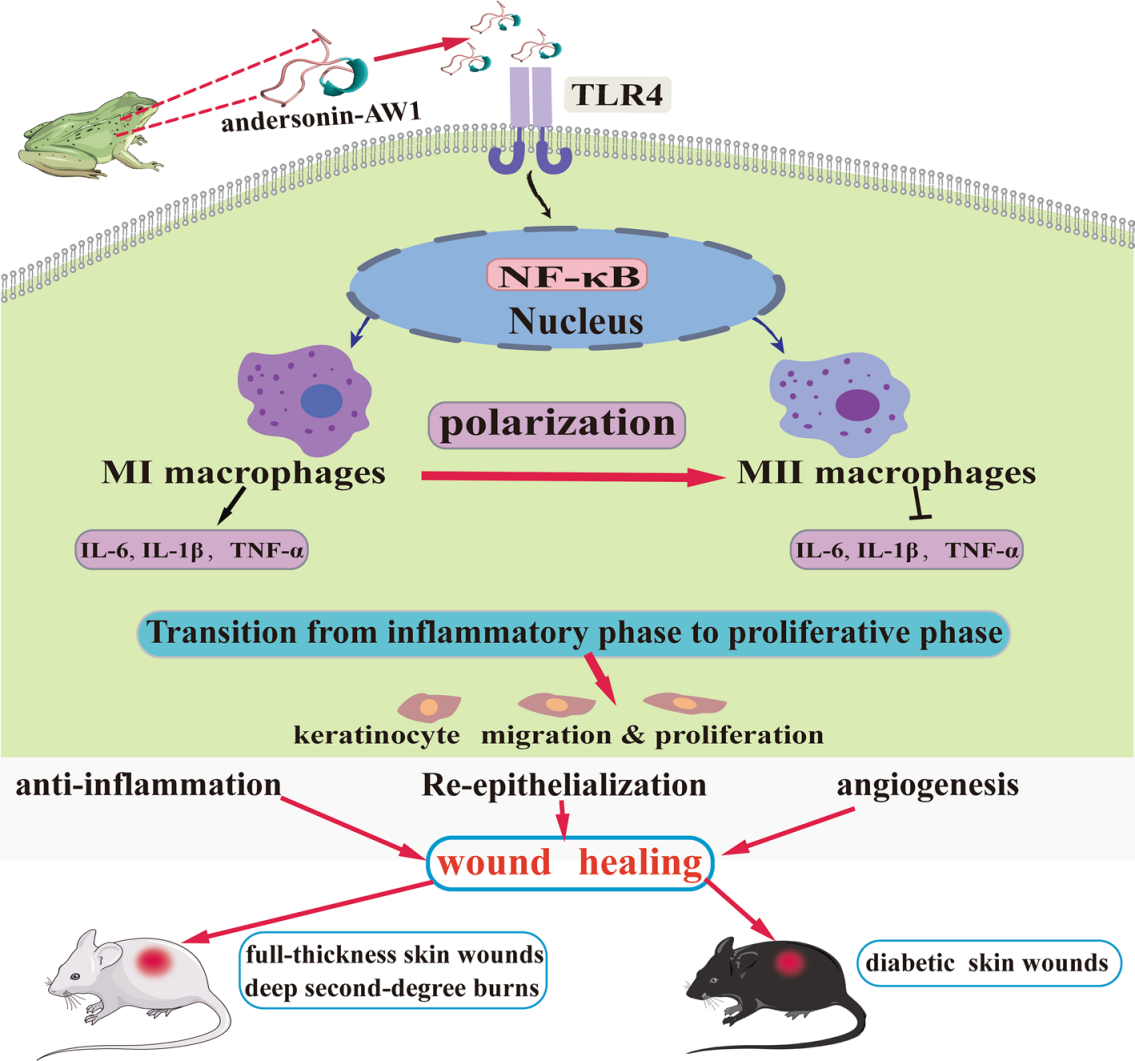

**Supplementary Information:**

The online version contains supplementary material available at 10.1186/s11658-024-00542-4.

## Background

As the largest human organ, the skin not only is the first line of defense against attack and pathogen invasion but also plays an important role in physiological processes [1, 2]. Skin structure integrity is crucial for its physiological functions [3]. However, skin is vulnerable to the external environment and disease, which can impair its structural integrity and functions. As a fundamental physiological process for maintaining skin integrity, wound healing involves the sophisticated regulation of multifunctional cells, cytokines, receptors, and signaling pathways to achieve spatially and temporally distinct cellular reorganization [4, 5]. Notably, macrophages are key regulators of immune responses during wound repair process and are considered as therapeutic targets for chronic wound healing [6]. TLR4 is a pattern recognition receptor that plays an important role in modulating immune response, and deficiency of TLR4 leads to decreased inflammation, increasing infection, and impaired reepithelialization in skin wounds in mice [7]. Moreover, TLR4 can regulate the macrophage inflammation via the NF-κB and MAPK signal pathway [8]; however, the function of TLR4 in modulating the macrophage NF-κB pathway in wound healing is poorly understood. Thus, using exogenous peptide as a probe to elucidate mechanisms with which TLR4 regulates inflammatory response of macrophages to accelerate healing will provide new therapeutic strategies for wound repair.

Wound healing is a precisely regulated physiological process; however, various factors can impede repair and lead to the development of chronic nonhealing wounds [9–11]. Although stem cell therapy, tissue engineering, and keratinocyte transplantation provide novel strategies for chronic wound healing, clinical treatment remains a significant challenge [12–15]. At present, the clinical therapeutic drugs for wound healing include small molecule compounds, growth factors, and wound dressings [16–19]. However, these treatments possess several limitations, including poor stability, difficult synthesis, and excessive wound repair, which extremely hamper their clinical application [20]. In contrast to small molecule compounds and growth factors, peptides exhibit higher activity, better affinity and selectivity, and lower toxicity [3, 16, 21]. Furthermore, the commercial market for peptide drugs is extensive, with more than 80 peptide drugs currently approved for clinical application [22–24]. The remarkable achievements made in the clinical application of peptide-based drugs highlight their considerable potential for further development [25]. However, despite these achievements, the development of prohealing peptide-based drugs remains in its infancy, and the underlying mechanisms of prohealing peptides remain investigated.

Amphibian-derived peptides not only demonstrate functional diversity and substantial clinical prospects but also usher in a new era in the development of peptide-based drugs for clinical application [21, 24, 26–31]. Although prohealing peptides exhibit high activity and broad market prospects, the discovery of peptide-based drug candidates with multifunctional activities is limited. Furthermore, prohealing agents based on bioactive peptides with antibacterial, antiinflammatory, and angiogenic activities provide a new strategy for chronic wound repair [26, 29]. AW1, a peptide obtained from *Oreochromis andersonii*, contains 72 amino acid residues, and the amino acid sequence of its mature peptide is ATNIPFKVHFRCKAAFC [32]. AW1 showed a great antimicrobial ability against *Staphylococcus aureus*, *Escherichia coli*, *Bacillus pyocyaneus*, and *Candida albicans* [32]. However, the function of AW1 in skin wound healing remains unknown.

Here, we provide solid evidence for the excellent prohealing effects of AW1, which showed inflammatory regulation (macrophage polarization), as well as promotion of reepithelialization, granulation regeneration, and angiogenic activities. Our research provides a promising multifunctional prohealing candidate agent for chronic skin wound healing, offers new insights into identification of the activities of amphibian-derived peptides, and emphasizes the critical role of exogenous small peptides in elucidating the mechanisms of human diseases.

## Materials and methods

### Peptide synthesis

AW1 peptide (ATNIPFKVHFRCKAAFC), fluorescein isothiocyanate (FITC)-labeled AW1, and scrambled peptide (ATVHNAAFIPFKFRCKC) were commercially synthesized by Wuhan Bioyeargene Biotechnology Co., Ltd. (Wuhan, China) with a purity greater than 95%. The purity of AW1 was detected by high-performance liquid chromatography (HPLC) according to a previous study [32].

### Cell culture

The mouse leukemic monocyte/macrophage cell line (RAW264.7, KCB200603YJ), human immortalized keratinocyte cell line (HaCaT, KCB200442YJ), and human umbilical vein endothelial cells (HUVECs, KCB2010233YJ) were obtained from the Chinese Academy of Sciences Kunming Cell Bank (Yunnan, China). HaCaT were seeded in Dulbecco’s modified Eagle medium/nutrient mixture F-12 (DMEM/F12, BI, Israel) supplemented with 10% (v/v) fetal bovine serum (FBS, Hyclone, USA) and antibiotics (100 units/mL penicillin and 100 units/mL streptomycin). HUVECs were cultured in high-glucose Dulbecco’s modified Eagle medium (DMEM, BI, Israel) with 10% FBS and 1% penicillin–streptomycin. The RAW264.7 cells were cultured in DMEM (BI, Israel) supplemented with inactivated 10% (v/v) FBS (Hyclone, USA) and antibiotics (100 units/mL penicillin and 100 units/mL streptomycin). All cells were cultured at 37 °C under a 5% CO_2_ atmosphere.

### Cell scratch assay

A cell scratch assay was performed according to a previous study [26] to explore the effects of AW1 and scrambled peptide on keratinocyte scratch repair. HaCaT (2 × 10^5^) were seeded in the 24-well plate and grown to confluency. Then, cell scratch was created by a 200-μL pipette tip and treated with vehicle [phosphate-buffered saline (PBS)], positive control [recombinant human basic fibroblast growth factor (rh-bFGF)], AW1 (1, 10, and 100 nM), and scrambled peptide (100 nM) for 24 h. The status of cell scratch was recorded by inverted microscope (Motic, AE2000, China) and calculated by Image J. All data were from three independent experiments performed in triplicate.

### Cell viability

The proproliferative activities of different concentrations of AW1 (1, 10, and 100 nM) and scrambled peptide (100 nM) on keratinocytes and macrophages were detected according to previous research [33]. Briefly, HaCaT and RAW264.7 (2.5 × 10^3^) were seeded in the 96-well plates. Then the cells were treated with PBS, AW1 (1, 10, and 100 nM), and scrambled peptide (100 nM) for 24 h, and MTS Cell Proliferation Assay Kit (Promega, USA) was used to detect cell viability. All data were from three independent experiments performed in triplicate.

### Cell migration

Transwell assay was performed to explore the effects of AW1 and scrambled peptide against keratinocyte migration following a previous study [34]. Keratinocytes (2 × 10^5^ cells/well) in the serum-free medium were seeded into the upper chamber of 24-well plates (Corning, USA) with 8-μm pore filters and treated with vehicle (PBS), different concentrations of AW1 (1, 10, and 100 nM), and scrambled peptide (100 nM). A total of 500 μL DMEM/F12  with 10% FBS was added to the lower chamber. After incubation for 24 h, the keratinocytes on the upper surface of the filter membranes were removed, and the migrated cells were stained with 0.1% crystal violet for 15 min. The migrated cells were recorded by inverted microscope. Finally, the stained cells were eluted by 33% glacial acetic acid, and absorbance was detected at 570 nm to indirectly reflect the number of migrated cells. Each experiment was performed three times independently in triplicate.

### Tube formation assay

Tube formation assay was performed by Matrigel basement membrane matrix (356234, Biosciences, San Jose, USA) according to a previous study [35]. Briefly, after thawing overnight at 4 °C, 50 μL Matrigel was added to 96-well plates and incubated at 37 °C to solidify. HUVECs (2 × 10^4^) were cultured in the 96-well plates and treated with vehicle (PBS) and different concentrations of AW1 (1, 10, and 100 nM) for 6 h. The tube formation was recorded by an inverted microscope and the tube length was randomly measured in five fields by ImageJ software. Each experiment was repeated three times independently.

### Experimental animals

Male Kunming mice and male C57BL/6 mice (20–24 g, 6–8 weeks) were purchased from Kunming Medical University (Kunming, China). All mice were fed individually in PVC cages under a 12-h light/12-h dark cycle. Before the experiment, the mice were adaptively cultivated for 1 week.

### Full-thickness skin wounds in Kunming mice

The therapeutic effects of AW1 (1, 10, and 100 nM) and scrambled peptide (100 nM) on full-thickness skin wounds in mice were determined according to a previous study [26]. After anesthetizing, the back hair of the mice was removed, and two equally large symmetrical wounds (10 mm) were created on the dorsal of each mouse using a punch. The mice were randomly divided into six groups, with five mice in each group. The wounds were topically treated with vehicle (PBS), recombinant human basic fibroblast growth factor (rh-bFGF, Beijing Bersee Science and Technology Co. Ltd., China, 100 ng/mL), different concentrations of AW1 (1, 10, and 100 nM), or scrambled peptide (100 nM) twice a day (20 μL per administration). Wound status was recorded every other day postsurgery until day 8, and wound area was quantified using ImageJ and analyzed using GraphPad Prism. Skin wound samples were acquired at days 4 and 8 postsurgery. Each experiment was performed three times independently.

### H&E staining

To explore the regeneration of skin wounds after treatment, H&E staining was performed as per a previous study [29]. Wound regeneration was recorded using a light microscope at the same magnification (×40). To evaluate the thickness of the neoepidermis and new granulation tissue in skin wounds of mice, five values were randomly measured in one field using ImageJ, and mean values were calculated and then analyzed by GraphPad Prism [3].

### Immunohistochemistry

To demonstrate the proliferation of epidermal cells and detect the inflammatory response, immunohistochemistry stain was performed according to a previous study [26]. The sliced tissues were incubated with primary antibodies, including rabbit anti-mouse interleukin-1β (IL-1β, Affinity; AF5103, China; 1:100), interleukin-6 (IL-6, Servicebio; GB11117, China; 1:600), tumor necrosis factor α (TNF-α, Servicebio; GB11188, China; 1:500), and Ki67 (Servicebio; GB11499, China; 1:500) at 4 °C for 12 h. Secondary antibodies (Servicebio; GB23303, China; 1:400) were used for incubation at 37 °C for 1 h, and diaminobenzidine staining (Biosharp, China) was performed. The positive staining of IL-1β, IL-6, TNF-α, and Ki67 was recorded by light microscopy (Primo Star, Zeiss, Germany) and measured by ImageJ software.

### Deep second-degree burns in mice

Deep second-degree burns were established in mice following a previous study [33]. Briefly, two deep second-degree burns were created on the dorsal of each mouse. Wounds were topically treated with vehicle (PBS), rh-bFGF (100 ng/mL), or different concentrations of AW1 (1, 10, and 100 nM) twice a day (20 μL per administration). Skin wound condition was documented on days 0, 4, 8, and 14 postoperation. Skin samples from the central area of the wounds were obtained on days 3, 4, 8, and 14 and used for histological analysis. Each experiment was repeated three times independently.

### Full-thickness skin wounds in diabetic mice

A type 2 diabetes C57BL/6 mouse model was established according to previous research [36]. Fasting blood glucose levels of mice were detected on days 3, 7, 14, 22, and 30 after the injection of streptozotocin (Solarbio, China). The mice with fasting blood glucose levels higher than 16.7 mmol/L were considered as type 2 diabetic. The therapeutic effects of AW1 (1, 10, and 100 nM) on diabetic mouse full-thickness skin wounds were determined according to a previous study [26]. In short, two symmetrical full-thickness wounds (10 mm) were created on the dorsal of type 2 diabetic mice. The diabetic mice with full-thickness skin wounds were randomly divided into five groups: diabetic control (PBS), rh-bFGF (100 ng/mL), and AW1 groups (1, 10, and 100 nM). Wild-type mice with full-thickness skin wounds were used as normal control (PBS). Wounds were topically treated with PBS, rh-bFGF, or different concentrations of AW1 twice a day. Wound condition was recorded on days 0, 4, 8, 12, and 18. Wound tissues were acquired on postoperative days 3, 8, 12, and 18. Each experiment was repeated three times independently.

### Enzyme-linked immunosorbent assay (ELISA)

To explore the effects of AW1 (1, 10, and 100 nM) on inflammatory factor expression in macrophages and chronic wounds, ELISA was performed following previous research [26, 34]. Briefly, macrophages (2 × 10^6^ cells/well) were seeded in 6-well plates and randomly treated with PBS (vehicle), lipopolysaccharide (LPS, 1 μg/mL), or different concentrations of AW1 for 24 h. The supernatants were collected, and the expression levels of proinflammatory cytokines, including TNF-α, IL-1β, and IL-6, were detected on the basis of instructions provided with the ELISA kits (NeoBioscience, Shanghai, China). The expression of TNF-α, IL-6, and IL-1β in chronic skin wound tissues (days 3, 8, and 12) were detected by ELISA kits (NeoBioscience, Shanghai, China). Each experiment was performed three times independently.

### Immunofluorescence

The wound tissues were blocked using 5% (w/w) goat serum containing 0.3% TritonX-100 for 1 h, then incubated with primary antibodies, including rat anti-mouse F4/80 (Abcam, ab6640, USA; 1:100), inducible nitric oxide synthase (iNOS, Abcam, ab178945, USA; 1:300), arginase (ARG)-1 (Affinity, DF6657, China; 1:200), CD31 (Affinity AF0077, China; 1:200), vascular endothelial growth factor (VEGF, Abcam, ab233693, USA; 1:500), and α-smooth muscle actin (α-SMA, Cell Signaling Technology, no. 19245, USA; 1:200), at 4 °C for 12 h, respectively. The wound tissues were then incubated with secondary antibodies, including goat anti-rabbit IgG Alexa Fluor^®^ 488 (Abcam, ab150077, USA; 1:200) and goat anti-rat IgG Alexa Fluor^®^ 647 (Abcam, ab150159, USA; 1:200), for 1 h at 37 °C, then stained with 4′,6-diamidino-2-phenylindole (DAPI). A confocal laser scanning microscope (Zeiss LSM800, Germany) was used for recording, with quantification of fluorescence intensity and statistical analysis performed using ImageJ and GraphPad Prism.

### Molecular docking

Molecular docking between AW1 and TLR4 was performed according to a previous study [37].

### Colocalization of TLR4 and AW1

Macrophages (2 × 10^3^ cells/well) were seeded in 12-well plates containing circular microscope cover glass (14 mm, Nest). After attachment, macrophages were treated with FITC-labeled AW1 (100 nM) for 1 h, and fluorescent staining was performed according to previous study [38]. Briefly, macrophages were incubated with TLR4 primary antibody (Affinity, China, 1:300) for 12 h at 4 °C, then incubated with secondary antibody (goat anti-rabbit IgG Fluor 594-conjugated, Affinity, China, 1:300) for 1 h at 37 °C. After DAPI staining and sealing, a confocal laser scanning microscope (Zeiss LSM800, Germany) was used for observation.

### Western blotting

Mouse macrophages (2 × 10^6^) were seeded in 6-well plates and treated with PBS, AW1 (1, 10, and 100 nM), LPS (1 μg/mL), or a TLR4 inhibitor (1 μg/mL) for 24 h. Macrophages and mouse skin tissue with deep second-degree burns (days 3 and 8) were lysed using cell lysate (RIPA:PMSF:phosphatase inhibitor of 100:1:1). The protein supernatants were collected after centrifugation (14,000*g*, 20 min, 4 °C) and quantified using the Bradford method (BCA protein analysis kit, Meilun, Dalian, China). Western blotting was performed to detect the effects of AW1 on the nuclear factor‐κB (NF-κB) signaling pathway and TLR4/NF-κB molecular axis according to previous studies [3, 39]. Primary antibodies, including GAPDH, Iκb, P-iκb, P65, and P-P65 (Affinity; China, 1:1 000), were used for western blotting according to the provided instructions. Each experiment was performed five times independently.

## Results

### AW1 inhibited excessive inflammation and promoted reepithelialization to accelerate full-thickness wound healing in mice

**Fig. 1:**
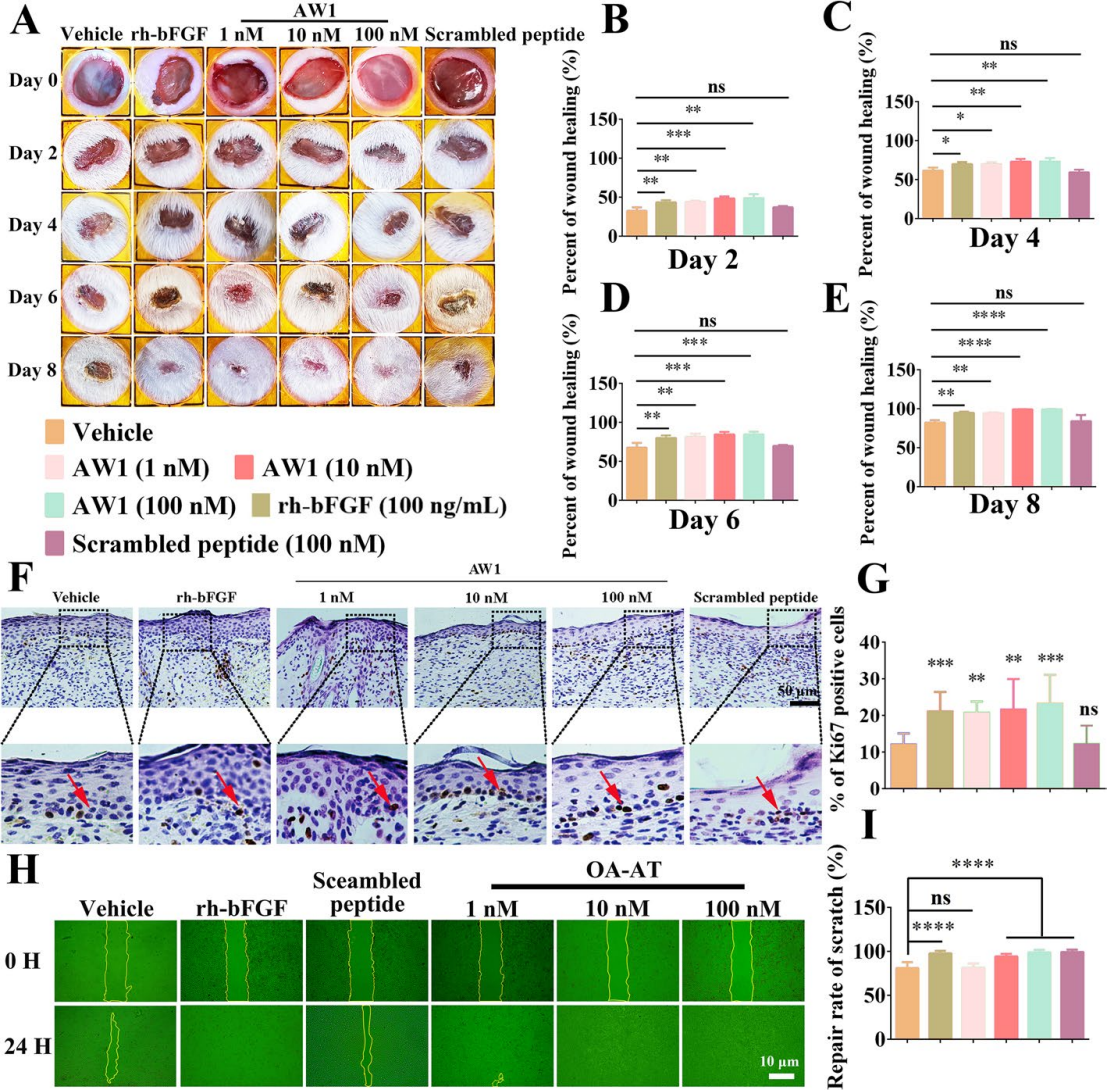
AW1 promoted reepithelialization to accelerate full-thickness wound repair and keratinocyte scratch wound healing. **A** Full-thickness wound status in mice under PBS, rh-bFGF, AW1 (1, 10, and 100 nM), and scrambled peptide treatment on days 0, 2, 4, 6, and 8. Peptides and rh-bFGF were dissolved in PBS to obtain AW1 (1, 10, and 100 nM), scrambled peptide (100 nM), and rh-bFGF (100 ng/mL) solutions. Each wound was treated with vehicle (20 µL, PBS), rh-bFGF (20 µL), different concentrations of AW1 (20 µL), or scrambled peptide (20 µL) twice a day from days 0–8, respectively. **B**–**E** Skin wound repair rate on days 0, 2, 4, 6, and 8. Data are expressed as mean ± standard error of the mean (SEM) from six mice (*n* = 6). **F** Expression of Ki67 in neoepidermis after PBS, rh-bFGF, AW1 (1, 10, and 100 nM), and scrambled peptide treatment on day 8 postoperation. Red arrows indicate positive staining, scale bar 50 μm. **G** Quantitative expression of Ki67 in mouse skin wounds on day 8. Data are expressed as mean ± standard error of the mean (SEM) from six mice (*n* = 6). **H** Representative images of keratinocyte scratch wound healing under PBS, rh-bFGF (100 ng/mL), AW1 (1, 10, and 100 nM), and scrambled peptide (100 nM) treatment at 0 h and 24 h, scale bar 10 μm. **I** Quantification of keratinocyte scratch repair rate under AW1 (1, 10, and 100 nM) treatment for 24 h. Data are expressed as mean ± standard error of the mean (SEM) from three independent experiments (*n* = 3). Ns, no significance; *P < 0.05, **P < 0.01, ***P < 0.001, and ****P < 0.0001 indicate statistically significant difference compared with vehicle

Amphibian-derived peptides are characterized with high activity, specificity, and functional diversity [3, 40]. Here, we found an excellent prohealing peptide AW1, whose chemical structure formula and the advanced structure are shown in Additional file 1: Fig. S1A–D. Moreover, AW1 was commercially synthesized, and the purity of AW1 was 95.57% (Additional file 1: Fig. S1E, F). AW1 exhibited remarkable prohealing properties in full-thickness skin wounds of mice (Fig. 1A). Notably, AW1 promoted wound repair in a concentration-dependent manner, reaching a healing rate of nearly 100% after 8 days of treatment at a concentration of 1 nM, which was significantly higher than that of the vehicle (82.01%) (Fig. 1B–E). However, the scrambled peptide (isotype control) did not exhibit any prohealing activity, suggesting that AW1 has structural specificity (Fig. 1B–E).

After treatment of skin wounds with vehicle and scrambled peptide, new epidermis and granulation tissue gradually formed, but there were obvious defects in the epidermis and thinner granulation tissue (Additional file 1: Fig. S2A). Compared with the vehicle, rh-bFGF treatment increased thickness of the neoepidermis and granulation tissue in the skin wounds (Additional file 1: Fig. S2B–E). Notably, AW1 (10 nM) treatment showed greater therapeutic effects, with thinner epidermis and granulation tissue on day 8 and similar structures to normal skin (Additional file 1: Fig. S2A–E).

The results of immunohistochemical staining showed that AW1 significantly suppressed the expression of proinflammatory factors compared with the vehicle and rh-bFGF (Additional file 1: Fig. S3A). Notably, in the AW1 (1 nM)-treated group, the expression level of IL-6, IL-1β, and TNF-α were only 0.70-, 0.62-, and 0.71-fold that of the vehicle group, respectively (Additional file 1: Fig. S3B–D). Furthermore, AW1 significantly promoted the expression of Ki67 in the neoepidermis (Fig. 1F, G). These results suggested that AW1 inhibited excessive inflammation and promoted reepithelialization, thus accelerating full-thickness wound healing.

### AW1 promoted the proliferation and migration of keratinocytes, macrophage proliferation, and tube formation of HUVEC

As shown in Fig. 1H–I, the scratch repair rate in the vehicle group was 80.7%, but it reached 97.5% in the rh-bFGF-treated group at 24 h. Interestingly, AW1 markedly promoted keratinocyte scratch repair, with a scratch repair rate of 98.9% in the AW1 (10 nM) group (Fig. 1H, I). Moreover, AW1 significantly promoted keratinocyte migration and proliferation (Additional file 1: Fig. S4A–C). As key regulators of the wound healing process, macrophages are considered as therapeutic targets for chronic wound healing [6, 41]. Significantly, AW1 also promoted macrophage proliferation (Additional file 1: Fig. S4D). The effect of AW1 on angiogenesis in vitro was detected by HUVEC tube formation (Additional file 1: Fig. S4E). Compared with vehicle, AW1 significantly facilitated the tube formation in a concentration dependent (Additional file 1: Fig. S4F). However, scrambled peptide had no promoting activity on keratinocyte or macrophage migration and proliferation nor on tube formation of HUVEC (Additional file 1: Fig. S4).

### AW1 regulated inflammation and facilitated skin regeneration in deep second-degree burns in mice

**Fig. 2:**
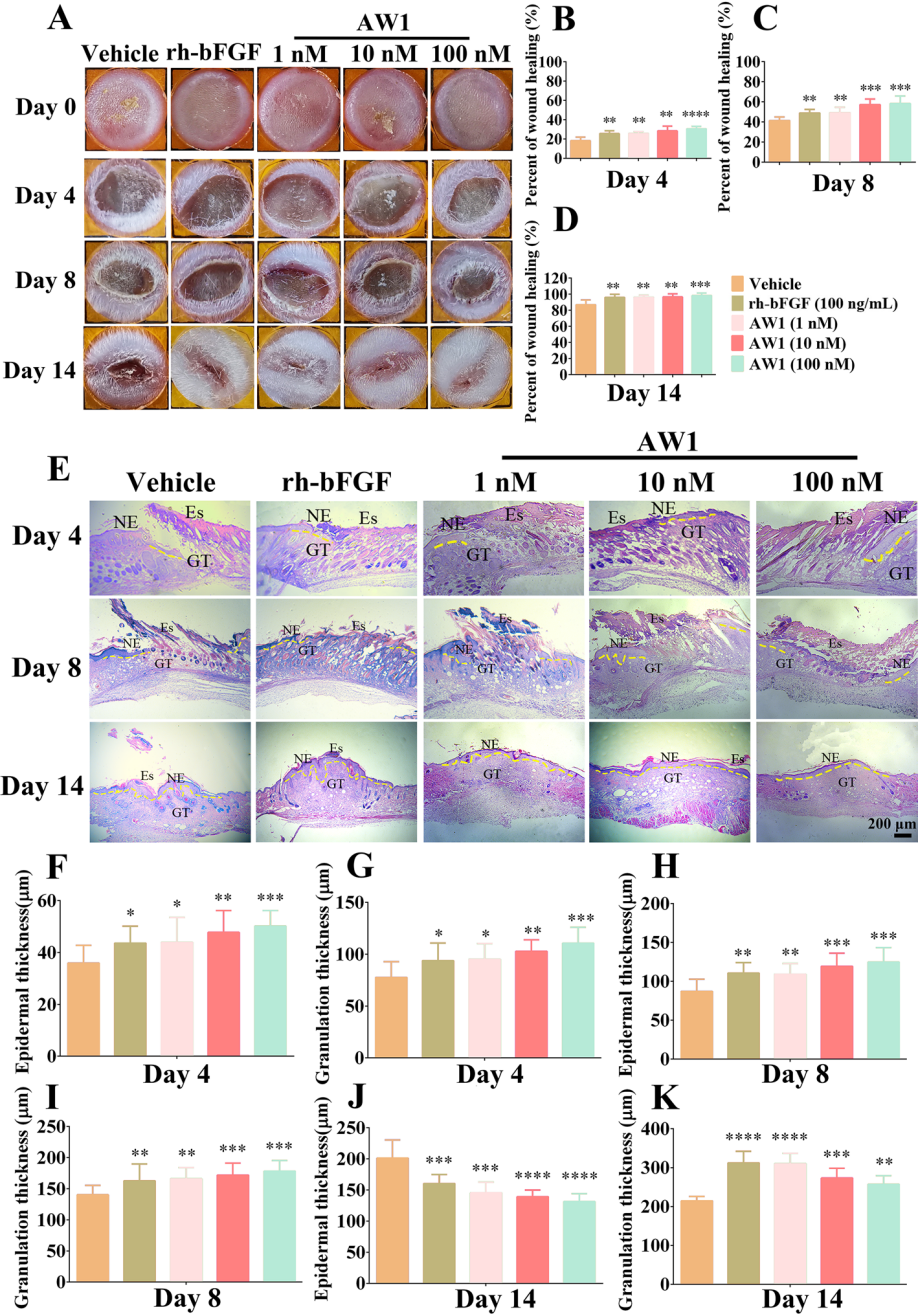
AW1 promoted reepithelialization and granulation tissue formation to accelerate deep second-degree burn healing in mice. **A** Representative images of deep-second degree burns in mice under PBS, rh-bFGF (100 ng/mL), or AW1 (1, 10, and 100 nM) treatment on days 0, 4, 8, and 14. Peptide and rh-bFGF were dissolved in PBS to obtain AW1 (1, 10, and 100 nM) and rh-bFGF (100 ng/mL) solutions. Each wound was treated with vehicle (20 µL, PBS), rh-bFGF (20 µL), or different concentrations of AW1 (20 µL) twice a day from days 0–14, respectively. **B**–**D** Quantification of wound repair rate on days 4, 8, and 14. E. H&E staining of deep-second degree burns on days 4, 8, and 14. Yellow dotted lines represent areas of neoepidermis; Es, eschar; NE, neoepidermis; GT, regenerated granulation tissue; scale bar 200 μm. **F**–**K** Quantification of neoepidermis and new granulation tissue thickness in deep-second degree burns on days 4, 8, and 14. All data are expressed as mean ± SEM from six mice (*n* = 6); *P < 0.05, **P < 0.01, ***P < 0.001, and ****P < 0.0001 indicate statistically significant difference compared with vehicle

As shown in Fig. 2A–D, AW1 significantly promoted the deep second-degree burns healing in a concentration-dependent manner. Notably, on day 14, the wound repair rate under AW1 (1 nM) treatment reached 96.1%, but only 86.8% in the vehicle group. H&E staining revealed the state of tissue regeneration of deep second-degree burns following the treatment of AW1 (Fig. 2E). The PBS-, rh-bFGF-, and AW1-treated skin wounds showed gradual generation of new epidermis and granulation tissue, with AW1 (100 nM) treatment exhibiting the best therapeutic effect on the neoepidermis and granulation tissue (Fig. 2F–K). On day 14 postinjury, the epidermal and granulation defects were completely repaired in the AW1- and rh-bFGF-treated groups, but not in the PBS-treated group (Fig. 2J, K). Moreover, the AW1-treated wounds showed thinner neoepidermis and granulation tissue than that in the rh-bFGF-treated group, with the reconstructed epidermis and granulation tissue more closely to normal skin.

Immunohistochemical staining showed that AW1 significantly promoted IL-6, IL-1β, and TNF-α expression on day 3 (Additional file 1: Fig. S5A–D). However, compared with the vehicle group, the expression of inflammatory factors in deep second-degree burns was significantly decreased under AW1 and rh-bFGF treatment for 8 days, and inflammatory response intensity was lower under AW1 (1 nM) treatment than rh-bFGF treatment (Additional file 1: Fig. S5E–H). Together, these results suggested that AW1 may regulate the inflammatory process during skin wound healing to accelerate wound healing.

### AW1 regulated macrophage polarization and NF-κB signaling pathway to promote the transition from inflammatory to proliferative phase

Macrophages play a crucial role in chronic wound healing, and their polarization is critical for the regulation of skin wound inflammation and the transition of inflammatory-to-proliferative phase [6, 42]. Immunofluorescence staining identified the M1 (F4/80/iNOS) and M2 macrophage (F4/80/Arg) phenotypes on days 3 and 8 postinjury (Additional file 1: Fig. S6A). Compared with the vehicle- and rh-bFGF-treated groups, AW1 significantly promoted the positive staining of M1 macrophages on day 3, while M2 macrophages showed no significant differences (Additional file 1: Fig. S6B, C). Notably, AW1 significantly decreased the positive staining of M1 macrophages but increased the positive staining of M2 macrophages in the skin wounds on day 8 (Additional file 1: Fig. S6D, E).

**Fig. 3:**
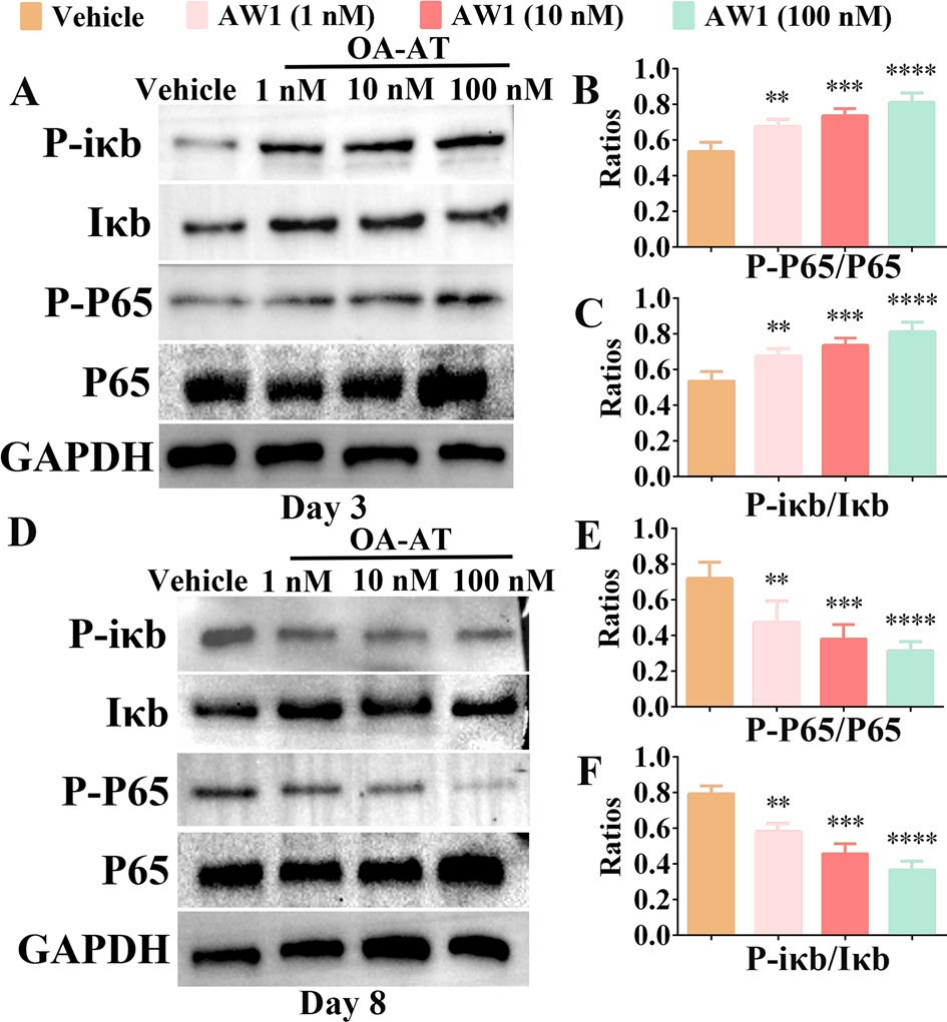
AW1 treatment regulated NF-κB signaling pathway activation in a concentration-dependent manner. **A** Effects of AW1 on NF-κB signaling pathway activation in deep second-degree burns at day 3 postinjury. **B**, **C** Quantification of relative expression of P65, P-P65, Iκb, and P-iκb in wound tissues at day 3. **D** Inhibitory effects of AW1 on NF-κB signaling pathway activation in deep second-degree burns at day 8 postinjury. **E**, **F** Quantification of relative expression of P65, P-P65, Iκb, and P-iκb in wound tissues at day 8. All data are expressed as mean ± SEM from five independent experiments (*n* = 5); **P < 0.01, ***P < 0.001, and ****P < 0.0001 indicate statistically significant difference compared with vehicle

The NF-κB signaling pathway is critical in regulating inflammation and macrophage polarization during skin wound healing [43]. Thus, we investigated the effects of AW1 on the NF-κB signaling pathway on days 3 and 8 postinjury. As shown in Fig. 3A–C, the expression levels of P-P65 and P-iκb significantly increased in a concentration-dependent manner  in the AW1-treated group on day 3 postinjury, indicating that AW1 promoted NF-κB signaling pathway activation. However, after 8 days of AW1 treatment, the activation of the NF-κB signaling pathway was significantly inhibited (Fig. 3D). The relative expression levels of P-P65 (P-P65/P65) and P-iκb (P-iκb/Iκb) were significantly higher in the vehicle group (0.72 and 0.79, respectively) than in the AW1 (1 nM) group (0.47 and 0.57, respectively; Fig. 3E–F). These results suggested that AW1 may regulate the NF-κB signaling pathway and macrophage polarization to modulate inflammation and promote inflammatory-to-proliferative phase transition and reepithelialization, thereby accelerating wound healing.

### AW1 promoted inflammation and inhibited excessive inflammation induced by LPS via the NF-κB signaling pathway

**Fig. 4:**
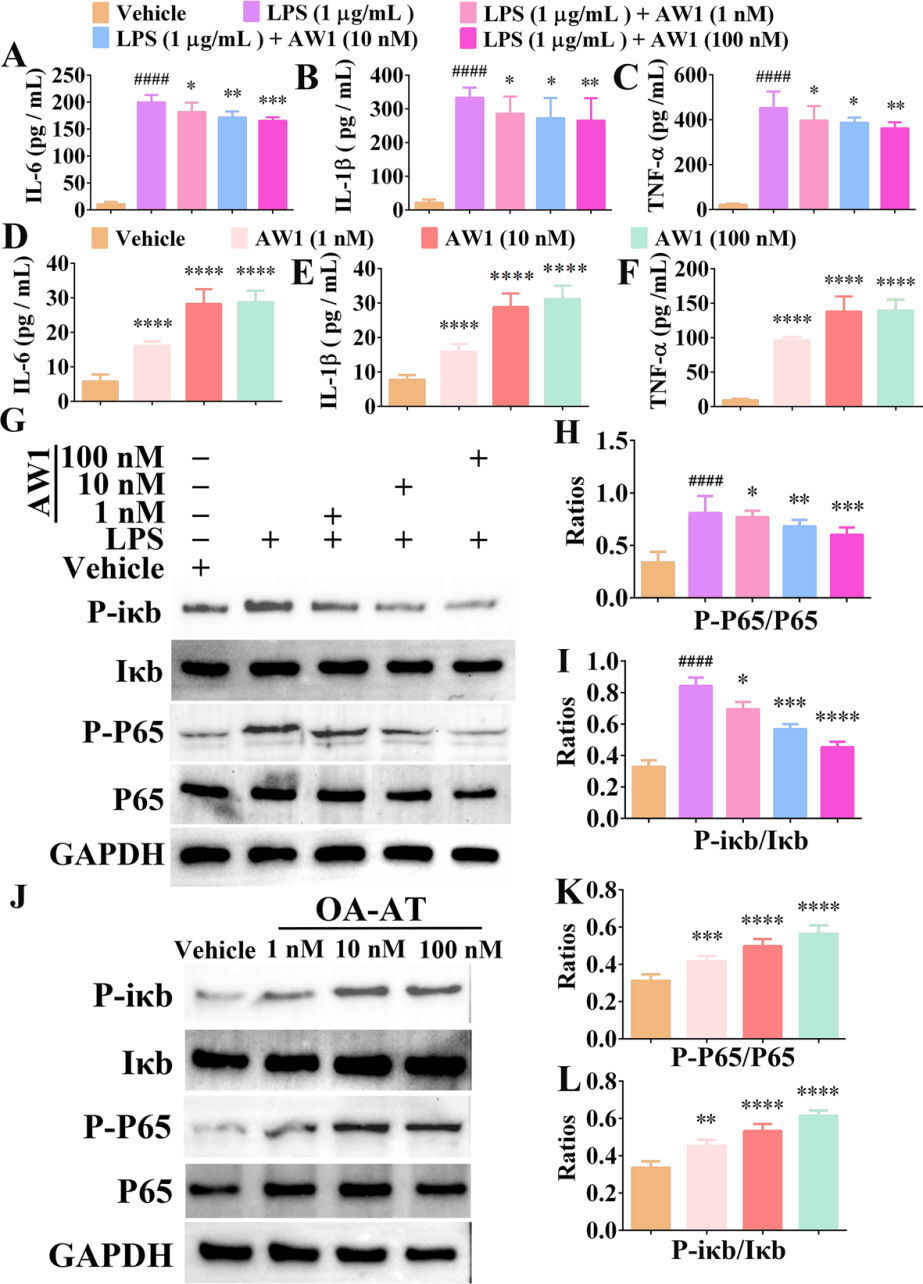
AW1 treatment regulated macrophage inflammatory responses via the NF-κB signaling pathway. **A**–**C** Inhibitory effects of AW1 (1, 10, and 100 nM) on excessive IL-6, IL-1β, and TNF-α expression after LPS simulation in macrophages. Data are expressed as mean ± SEM from three independent experiments (*n* = 3). **D**–**F** Effects of AW1 (1, 10, and 100 nM) on IL-6, IL-1β, and TNF-α expression in macrophages. Data are expressed as mean ± SEM from three independent experiments (*n* = 3). **G** Western blotting images of P-P65, P65, Iκb, and P-iκb in macrophages under PBS, LPS (1 μg/mL), or AW1 (1, 10, and 100 nM) treatment for 24 h. **H**–**I** Quantification of relative expression of P65, P-P65, Iκb, and P-iκb. Data are expressed as mean ± SEM from five independent experiments (*n* = 5). **J** Concentration-dependent promoting effects of AW1 on NF-κB signaling pathway activation. **K**–**L** Quantification of relative expression of P65, P-P65, Iκb, and P-iκb. Data are expressed as mean ± SEM from five independent experiments (*n* = 5). ^####^*P* < 0.0001 indicates statistically significant difference compared with LPS, *P < 0.05, **P < 0.01, ***P < 0.001, and ****P < 0.0001 indicate statistically significant difference compared with vehicle

To investigate the molecular mechanism underlying the regulation of AW1 on macrophage inflammation, we next explored the effects of AW1 on macrophage inflammatory factor expression and the NF-κB signaling pathway. AW1 significantly inhibited the expression of IL-6, IL-1β, and TNF-α in macrophages induced by LPS, indicating its inhibitory activity against excessive inflammation (Fig. 4A–C). Moreover, AW1 promoted the expression of IL-6, IL-1β, and TNF-α in a concentration-dependent manner (Fig. 4D–F). LPS stimulation significantly activated the NF-κB signaling pathway, while AW1 significantly inhibited P-P65 and P-iκb expression in a concentration-dependent manner, suggesting that AW1 inhibited NF-κB signaling pathway activation to suppress excessive inflammation (Fig. 4G–I). Interestingly, AW1 activated the NF-κB signaling pathway in a concentration-dependent manner, with AW1 (1 nM) treatment increasing phosphorylated P65 and Iκb expression by 1.32- and 1.36-fold that of the vehicle, respectively (Fig. 4J–L). These findings indicated that AW1 regulated the NF-κB signaling pathway to promote inflammatory factor expression and inhibit excessive inflammation induced by LPS stimulation, consistent with the results at the animal level.

### AW1 regulated macrophage inflammation via TLR4

**Fig. 5:**
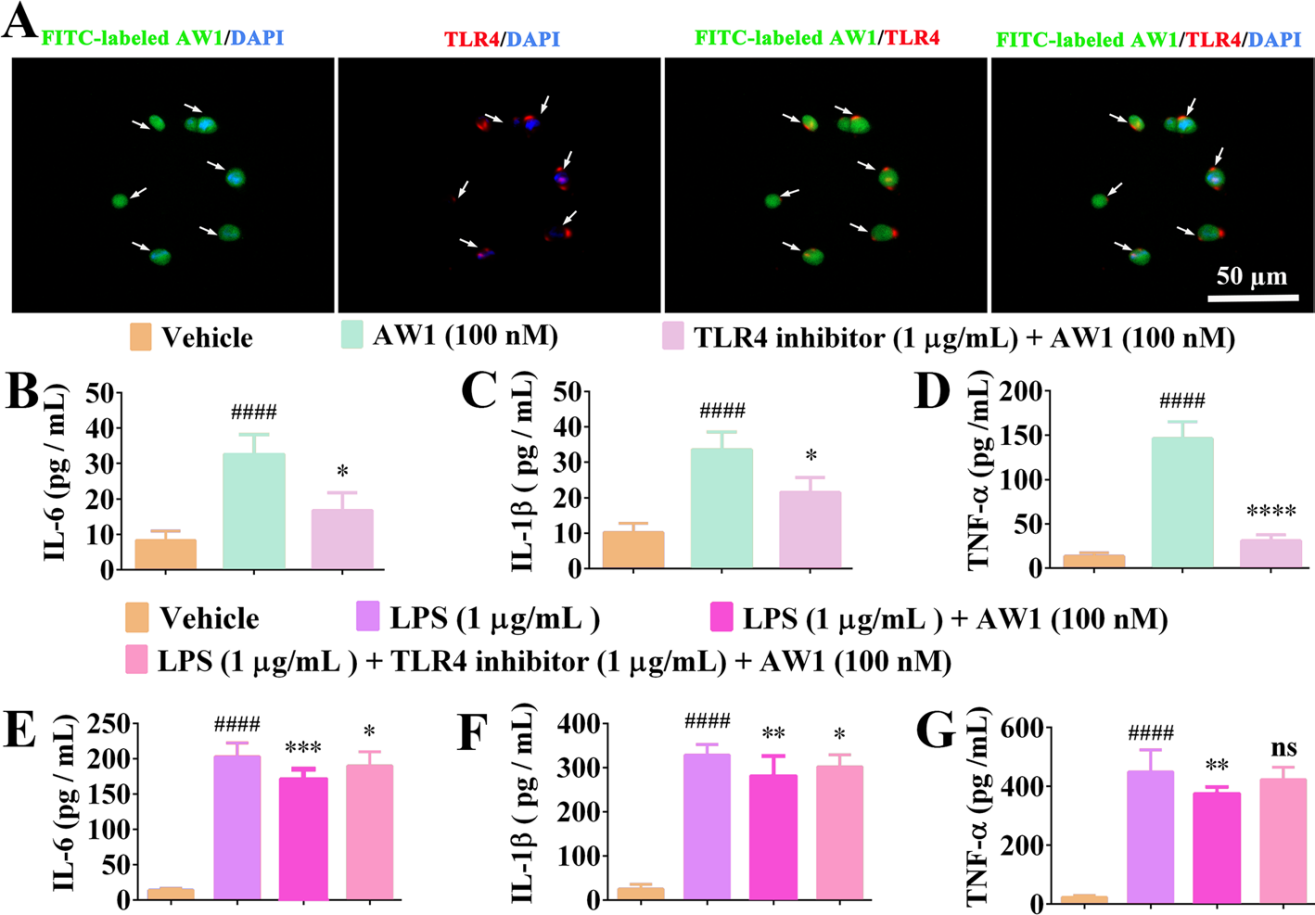
AW1 regulated IL-6, IL-1β, and TNF-α expression in macrophages via TLR4. **A** Colocalization of TLR4 and AW1 in macrophages under FITC-labeled AW1 treatment for 1 h; green fluorescence for FITC-labeled AW1, red fluorescence for TLR4, and DAPI blue fluorescence for nuclei; white arrows indicate binding of TLR4 and AW1; scale bar 50 µm. **B**–**D** Effects of AW1 (100 nM) on IL-6, IL-1β, and TNF-α expression after treatment with TLR4-specific inhibitor in macrophages. **E**–**G** Effects of AW1 (100 nM) on IL-6, IL-1β, and TNF-α expression in macrophages stimulated by LPS (1 µg/mL) after specific inhibition of TLR4 receptor. All data are expressed as mean ± SEM from three independent experiments (*n* = 3); ^####^*P* < 0.0001 indicates statistically significant difference compared with vehicle, *P < 0.05, **P < 0.01, ***P < 0.001, and ****P < 0.0001 indicate statistically significant difference compared with LPS

Extracellular molecules typically exert their biological functions by entering cell or extracellular receptors [44]. As shown Additional file 1: Fig. S7A, B, AW1 (gold) was able to bind to the TLR4 surface (white) through hydrogen bonding, with a binding free energy of −31 kcal/mol (Additional file 1: Table S1). More importantly, colocalization of AW1 and TLR4 directly demonstrated that green-fluorescence-labeled AW1 could bind to red-fluorescence-labeled TLR4 in the macrophages (Fig. 5A). Furthermore, TLR4-specific inhibitor significantly inhibited the proinflammatory factor expression activity of AW1 (100 nM) (Fig. 5B–D). AW1 suppressed the expression of excessive inflammatory factors induced by LPS, but its activity was significantly suppressed by the TLR4 specific inhibitor (Fig. 5E–G). These results suggested AW1 regulated macrophage inflammation by directly bounding to TLR4.

### AW1 regulated macrophage inflammation via TLR4/NF-κB molecular axis

**Fig. 6:**
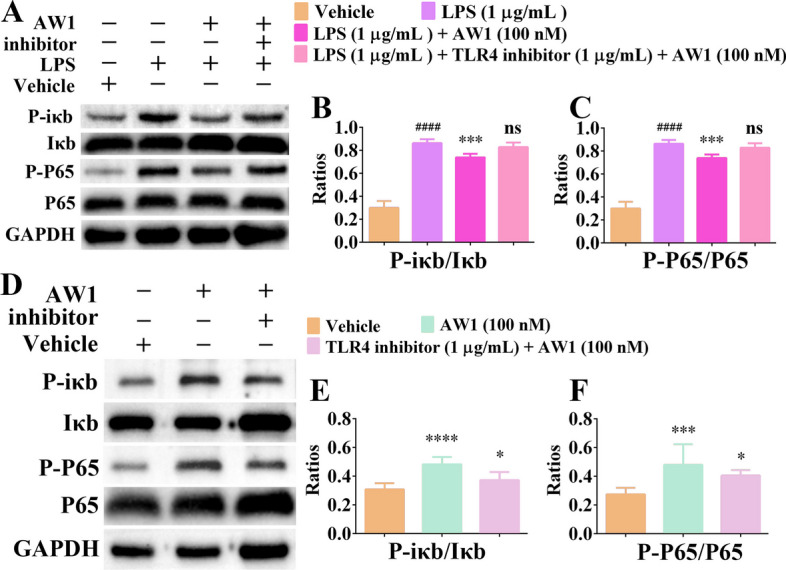
AW1 regulated macrophage inflammation via the TLR4/NF-κB molecular axis. **A** Western blotting images showing inhibitory effects of AW1 (100 nM) on NF-κB signaling pathway activation in macrophages induced by LPS. TLR4-specific inhibitor significantly suppressed AW1 activity. **B**, **C** Quantification of relative expression of Iκb, P-iκb, P65, and P-P65. **D** Effects of AW1 (100 nM) on NF-κB signaling pathway activation in macrophages after treatment with TLR4-specific inhibitor. **E**, **F** Quantification of relative expression of Iκb, P-iκb, P65, and P-P65. All data are expressed as mean ± SEM from five independent experiments (*n* = 5); ^####^*P* < 0.0001 indicates statistically significant difference compared with vehicle, *P < 0.05, ***P < 0.001, and ****P < 0.0001 indicate statistically significant difference compared with LPS

Our results show that AW1 regulated macrophage inflammation via TLR4. Notably, the NF-κB signaling pathway is a critical downstream pathway for TLR4 regulation of the inflammatory response [41, 45]. Thus, we speculated that AW1 regulated macrophage inflammation via the TLR4/NF-κB molecular axis. To test this hypothesis, western blotting was performed to explore whether AW1 affected macrophage inflammation via the TLR4/NF-κB molecular axis (Fig. 6A,D). LPS significantly promoted NF-κB signaling pathway activation, whereas AW1 inhibited the activation of NF-κB signaling pathway induced by LPS (Fig. 6B–C). TLR4-specific inhibitor significantly suppressed the effects of AW1 on suppressing NF-κB signaling pathway activation induced by LPS (Fig. 6B–C). Moreover, the promoting effect of AW1 on the NF-κB signaling pathway activation was inhibited by the TLR4-specific inhibitor (Fig. 6E–F). These results suggested that AW1 regulates macrophage inflammation via the TLR4/NF-κB molecular axis.

### AW1 regulated inflammation and promoted reepithelialization and angiogenesis to accelerate diabetic skin wound healing in mice

**Fig. 7:**
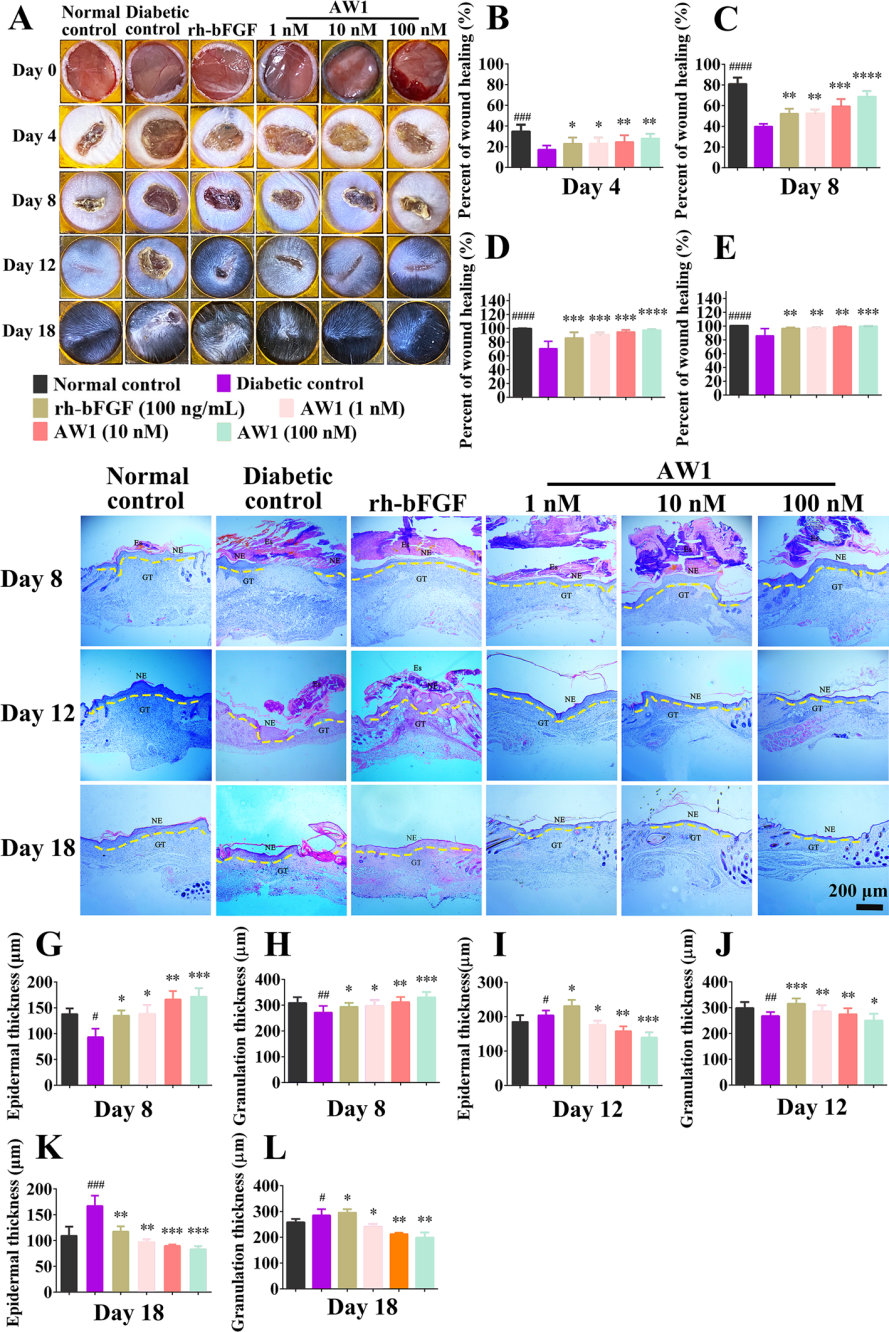
AW1 promoted reepithelialization to accelerate diabetic skin wound healing in mice. **A** Representative images of full-thickness skin wounds in diabetic mice under PBS, rh-bFGF (100 ng/mL), or AW1 (1, 10, and 100 nM) treatment on days 0, 4, 8, 12, and 18. **B**–**E** Quantification of wound repair rate on days 4, 8, 12, and 18. **F** H&E staining of full-thickness skin wounds in diabetic mice on days 8, 12, and 18. Yellow dotted lines represent areas of neoepidermis; Es, eschar; NE, neoepidermis; GT, regenerated granulation tissue; scale bar 200 μm. **G**–**L** Quantification of neoepidermis and new granulation tissue thickness on days 8, 12, and 18. All data are expressed as mean ± SEM from six mice (*n* = 6); ^#^*P* < 0.05, ^##^*P* < 0.01, and ^###^*P* < 0.001 indicate statistically significant difference compared with normal control; *P < 0.05, **P < 0.01, ***P < 0.001, and ****P < 0.0001 indicate statistically significant difference compared with diabetic control

The above results demonstrated that AW1 showed great therapeutic potential for chronic wound healing. Therefore, full-thickness skin wounds in diabetic mice (Additional file 1: Table S2) were performed to assess the therapeutic activity of AW1. Normal mouse skin wounds treated with PBS had largely healed on day 12 postinjury, whereas the healing of skin wounds in type 2 diabetic mice was obviously impaired (Fig. 7A). Notably, AW1 significantly promoted chronic skin wound healing in a concentration-dependent manner (Fig. 7B–E). The wound repair rate reached 99.5% after 18 days of AW1 (100 nM) treatment, better than the therapeutic effect achieved by rh-bFGF (96.3%), while the wound healing rate was only 85.8% in PBS-treated diabetic mice (Fig. 7E).

H&E staining showed that the neoepidermis had reconstructed and covered the skin wounds in normal mice on day 18 postinjury, whereas the diabetic mouse skin wounds still exhibited epidermal defects (Fig. 7F). In contrast, the new generated epidermis and granulation tissue had fully covered the wound with the treatment of AW1 (1 nM) on day 12 postinjury. More importantly, the thickness of the newly generated epidermis and granulation tissue in the AW1 (100 nM)-treated group was significantly thinner than that observed in the rh-bFGF-treated group, and no hyperplasia was observed (Fig. 7F–L).

**Fig. 8:**
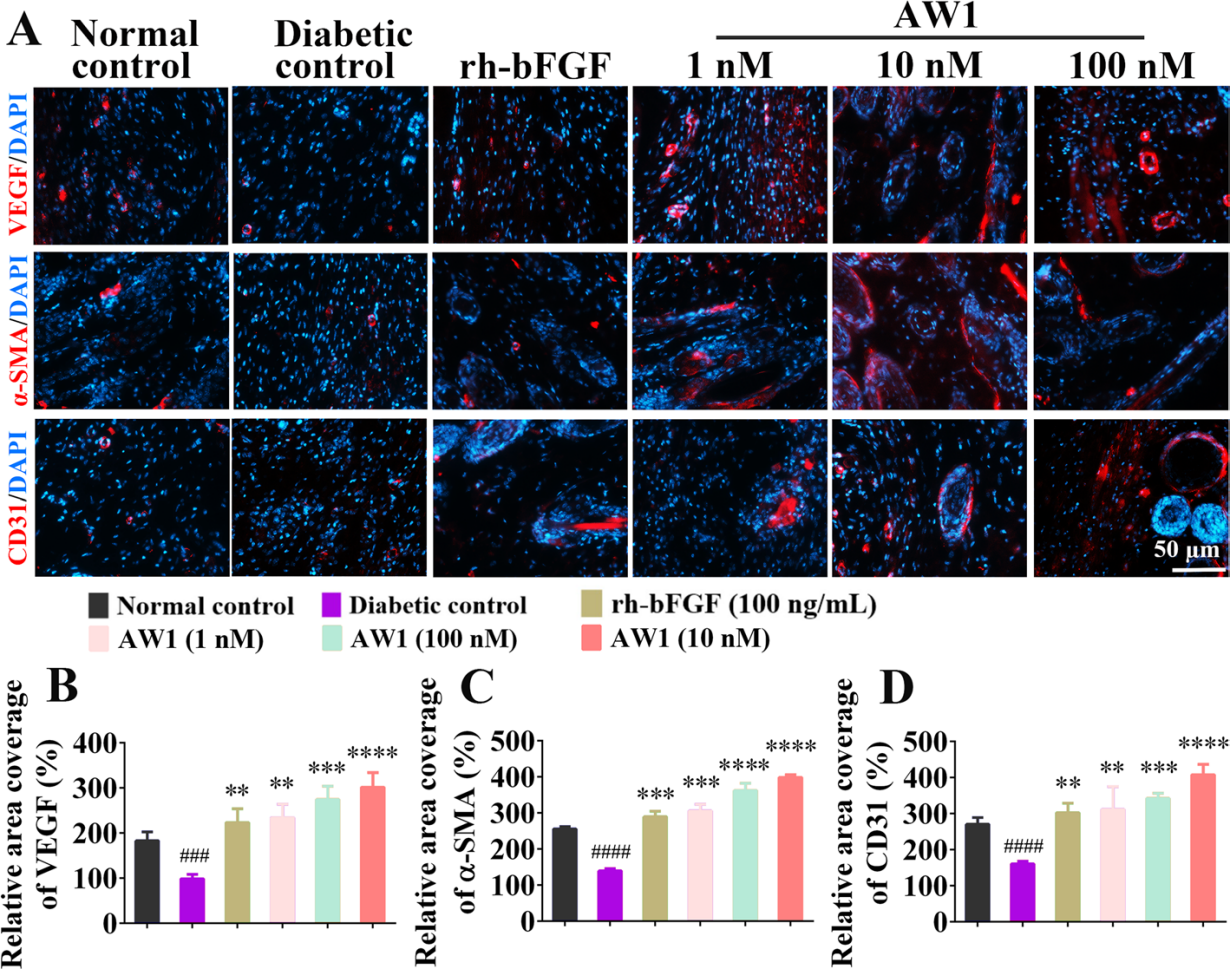
AW1 promoted angiogenesis to accelerate diabetic skin wound healing. **A** Images of immunofluorescence staining of VEGF, α-SMA, and CD31 in diabetic skin wounds on day 18. Red fluorescence for VEGF, α-SMA, and CD31, and DAPI blue fluorescence for nuclei; scale bar 50 µm. **B** Quantification of VEGF expression. **C** Quantification of α-SMA expression. **D** Quantification of CD31 expression. All data are expressed as mean ± SEM from six mice (*n* = 6); ^###^*P* < 0.001 and ^####^*P* < 0.0001 indicate statistically significant difference compared with normal control; **P < 0.01, ***P < 0.001, and ****P < 0.0001 indicate statistically significant difference compared with diabetic control

Compared with the normal mice, inflammatory response intensity was significantly higher in the wounds of diabetic mice, demonstrating the successful construction of chronic wounds (Additional file 1: Fig. S8). As shown in Additional file 1: Fig. S8A–C, compared with the diabetic control, AW1 treatment significantly upregulated the expression of IL-6, IL-1β, and TNF-α on day 3 but inhibited the excessive inflammation in diabetic wounds with prolonged treatment (days 8 and 12). These results were consistent with the changes of inflammatory response during the repair of deep second-degree burn, suggesting that AW1 may regulate macrophage polarization and NF-κB to affect inflammation and promote wound repair process. Macrophage polarization (M1–M2 phenotype) could not only regulate the inflammation during wound but also promote vascular regeneration. Interestingly, the immunofluorescence staining revealed that the expression of VEGF, α-SMA, and CD31 in the reconstructed wound tissues after the treatment of AW1 were significantly higher than that in the diabetic control and rh-bFGF groups (Fig. 8A–D).

## Discussion

Amphibian-derived prohealing peptides provide new intervention strategies for chronic nonhealing wounds, especially peptide-based multifunctional prohealing agents, which provide new clues for the development of peptide-based drugs for clinical wound treatment [3, 26, 29]. However, despite their potential, candidate peptides with multifunctional activities remain rare. In the current study, we demonstrated that AW1, a previously reported antimicrobial peptide [32], exhibits excellent therapeutic effects on acute and diabetic skin wounds in mice. Compared with previously identified prohealing peptides, such as AH90, Tylotoin, OA-GL21, OM-LV20, OA-GL12, OA-FF10, Ot-WHP, RL-QN15, OA-GP11 dimer, OA-GL17d, and OA-RD17 [3, 33, 34, 46–54], AW1 exhibited antibacterial properties [32] as well as the ability to regulate inflammation, promote angiogenesis, and accelerate wound healing, indicating its greater potential for clinical application. Our findings not only provide a promising multifunctional prohealing drug candidate for chronic nonhealing skin wounds but also offer new insights into the identification of peptide functions and the development of peptide-based drugs for clinical treatment.

Amphibian-derived peptides with immunomodulatory properties have shown great potential for wound healing and are considered promising candidates in this field [52]. However, most previous studies have only explored the signaling pathway activation or immune cell changes modulated by these peptides [3, 34, 52], and the mechanism by which they regulate immune responses to accelerate wound healing at different stages remains poorly understood. In the early stage of wound healing, the expression of proinflammatory cytokines not only prevent infection but also recruit other inflammatory cells to secrete growth factors and cytokines, which promoted the activation and migration of functional cells to accelerate wound repair [55, 56]. However, persistent inflammation will impede the skin wounds and cause nonhealing wounds. Our results show that AW1 significantly promoted the expression of IL-6, IL-1β, and TNF-α at the early stage of wound healing but inhibited excessive inflammation in the proliferative stage of wound healing. Thus, we hypothesize that AW1 might regulate inflammation at different phases and promote the wound healing process to accelerate wound repair. Interestingly, our in vitro assay results indicate that AW1 could promote macrophage inflammatory factor expression and inhibit the excessive inflammation induced by LPS. These results indicate that there may be a complex mechanism with which AW1 modulates immune response and macrophage inflammation to accelerate wound healing.

Macrophages are considered as potential therapeutic targets for chronic skin wounds [6]. Moreover, the NF-κB signaling pathway and macrophage polarization are critical for modulating inflammation during skin wound healing [57, 58]. Our results indicate that AW1 promotes the activation of the NF-κB signal pathway and macrophage polarization to M1 phenotype at the early stage of inflammation. However, AW1 inhibited the NF-κB signal pathway and promoted the macrophage polarization from M1 phenotype to M2 phenotype in the proliferative phase. Importantly, macrophage polarization from M1 to M2 phenotype can promote the transition from inflammatory phase to proliferative phase and angiogenesis [42]. Furthermore, the NF-κB signaling pathway not only regulates macrophage polarization but also plays an important role in facilitating the transition from the inflammatory phase to the proliferative phase and accelerating wound healing [59]. Thus, AW1 may regulate the NF-κB signal pathway to modulate macrophage polarization and promote the transition from inflammation to proliferation during wound healing. Interestingly, AW1 activated the macrophage NF-κB signaling pathway to promote the expression of inflammatory factors, while suppressing LPS induced macrophage NF-κB signaling pathway activation and excessive inflammation. However, the mechanism with which AW1 regulates NF-κB signaling pathways to inhibit LPS-induced excessive inflammatory responses remains unclear.

Extracellular molecules typically exert their biological functions by entering cells or extracellular receptors [44]. TLR4 is a pattern recognition receptor, which regulates the downstream NF-κB signaling pathway to affect macrophage polarization and inflammatory response [60]. Moreover, TLR4 is critical for skin wound healing, and TLR4-deficient mice show a significant delay in wound healing and lower inflammatory response [7]. Our results show that AW1 regulated NF-κB signaling pathway and macrophage polarization during wound healing and then promoted skin wound healing. More importantly, AW1 regulated the macrophage NF-κB signaling pathway and inflammation by directly binding to TLR4 in the macrophage extracellular region. LPS could also regulate the macrophage NF-kB signaling pathway and immune responses by binding to TLR4. Therefore, we speculate that AW1 may competitively bind to TLR4 with LPS and then lead to changes in TLR4 conformation, thereby significantly reducing the binding of LPS to TLR4 and inhibiting the excessive inflammatory response induced by LPS. Our study revealed the intracellular signaling pathways (TLR4/NF-κB) regulated by exogenous peptide AW1, providing new insights into the mechanisms of prohealing peptides in skin wound treatment.

Chronic nonhealing wounds are caused by various factors, such as infection, excessive inflammation, impeded angiogenesis, and impaired cell function [61, 62]. Our results suggest that AW1 may modulate macrophage polarization and inflammation to promote the transition from the inflammatory to the proliferative phase during skin wound healing. The transition from the inflammatory to the proliferative phase is critical for chronic nonhealing wounds [63]. Moreover, different macrophage phenotypes exhibit different biological functions, and macrophage polarization from the M1 to M2 phenotype can suppress excessive inflammation and promote angiogenesis [42, 64, 65]. Notably, AW1 significantly promoted reepithelialization and angiogenesis to accelerate diabetic skin wound healing in mice, indicating that AW1 may regulate the NF-κB signaling pathway by TLR4 to affect macrophage polarization and inflammation. On the basis of our previously reported antibacterial activity of AW1 [32], and the current findings that it regulates macrophage polarization and inflammation and promotes reepithelialization and angiogenesis, it is highlighted that AW1 is a promising multifunctional candidate drug for the treatment of chronic nonhealing skin wounds.

It should be noted that there are significant differences between human and mouse skin. To better determine the therapeutic effect of AW1 on human skin, the prohealing ability of AW1 should be firstly tested on porcine skin wound repair and then explored in ex vivo skin wounds in human. Moreover, it is important to reveal the mechanism of AW1-regulating macrophage inflammation and inflammatory response at different stages of wound healing through TLR4 in further studies. In particular, whether and how AW1 regulates inflammation by competitively binding to TLR4 with LPS may provide clues that AW1 exhibits antibacterial activity and regulates inflammation to promote wound healing.

## Conclusions

We not only identified AW1 as a promising multifunctional prohealing drug candidate for chronic nonhealing skin wounds but also used it as a peptide probe to identify AW1 as an immunomodulatory agent that may regulate the macrophage NF-κB signaling pathway by binding to TLR4 in competition with LPS.

## Supplementary Information

**Additional file 1: Figure S1.** Chemical structure formula and the advanced structure of AW1. **Figure S2.** AW1 promoted reepithelialization to accelerate full-thickness wound repair. **Figure S3.** AW1 inhibited excessive inflammation in full-thickness wound. **Figure S4.** AW1 promoted proliferation and migration of keratinocyte, macrophage proliferation, and tube formation of HUVEC. **Figure S5.** AW1 regulated inflammatory response intensity changes in deep-second degree burns. **Figure S6.** AW1 regulated macrophage polarization and promoted macrophage transition from MI to MII phenotype. **Figure S7.** Representative images of molecular docking between TLR4 and AW1. **Figure S8.** AW1 inhibited excessive inflammation in diabetic skin wound healing. **Table S1.** TLR4-AW1 binding free energy. **Table S2.** Fasting blood glucose of modeled diabetic mice on different days.

## Data Availability

The datasets used and/or analyzed in the current study are available from the corresponding author upon reasonable request.

## Abbreviations

rh-bFGF: Recombinant human basic fibroblast growth factor
FBS: Fetal bovine serum
PBS: Phosphate-buffered saline
LPS: Lipopolysaccharide
H&E: Hematoxylin and eosin
TNF-α: Tumor necrosis factor alpha
IL-1β: Interleukin-1β
IL-6: Interleukin-6
iNOS: Inducible nitric oxide synthase
ARG: Arginase
TLR4: Toll-like receptor 4
NF-κB: Nuclear factor‐kappa B
