# Correction: The direct binding of bioactive peptide Andersonin‑W1 to TLR4 expedites the healing of diabetic skin wounds

**DOI:** 10.1186/s11658-026-00878-z

**Published:** 2026-02-18

**Authors:** Chao Li, Yuxin Xiong, Zhe Fu, Yuxin Ji, Jiayi Yan, Yan Kong, Ying Peng, Zeqiong Ru, Yubing Huang, Yilin Li, Ying Yang, Li He, Jing Tang, Ying Wang, Xinwang Yang

**Affiliations:** 1 Department of Anatomy and Histology & Embryology, Faculty of Basic Medical Science, Kunming Medical University, Yunnan, 650500 Kunming China; 2 Key Laboratory of Chemistry in Ethnic Medicinal Resources & Key Laboratory of Natural Products Synthetic Biology of Ethnic Medicinal Endophytes, State Ethnic Affairs Commission & Ministry of Education, School of Ethnic Medicine, Yunnan Minzu University, Yunnan, 650504 Kunming China; 3 Department of Biochemistry and Molecular Biology, Faculty of Basic Medical Science, Kunming Medical University, Yunnan, 650500 Kunming China; 4 Department of Endocrinology, Affiliated Hospital of Yunnan University, Yunnan, 650021 Kunming China; 5 Department of Dermatology, First Affiliated Hospital of Kunming Medical University, Yunnan, 650032 Kunming China

**Correction: Cellular & Molecular Biology Letters (2024) 29:24**

10.1186/s11658-024-00542-4

In this article [1], the wrong figure appeared as Fig. 1, S5 and S6; the correct figures should have appeared as shown below.

**Correct** **Figure 1**:

**Fig. 1:**
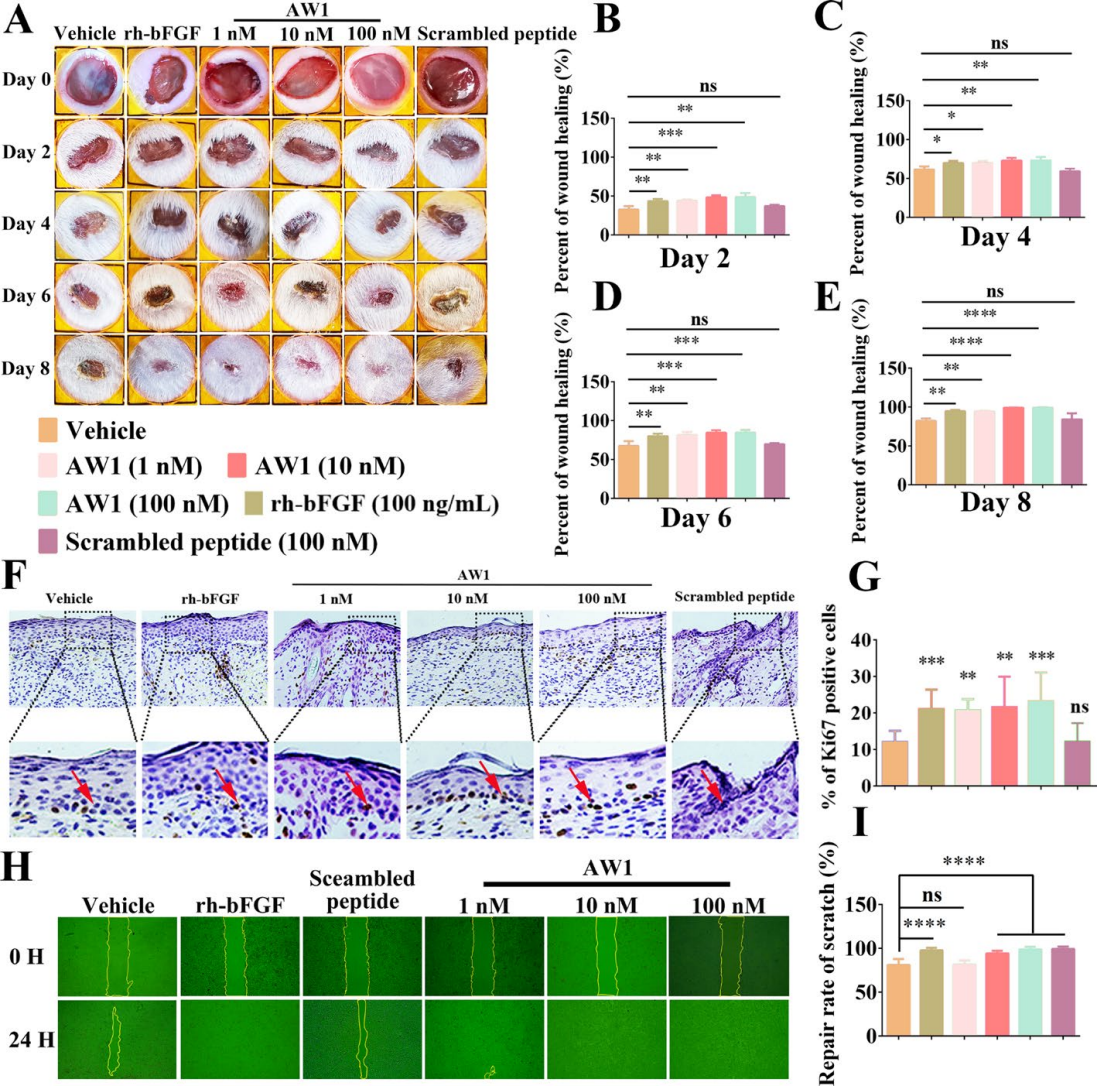
AW1 promoted reepithelialization to accelerate full-thickness wound repair and keratinocyte scratch wound healing. **A** Full-thickness wound status in mice under PBS, rh-bFGF, AW1 (1, 10, and 100 nM), and scrambled peptide treatment on days 0, 2, 4, 6, and 8. Peptides and rh-bFGF were dissolved in PBS to obtain AW1 (1, 10, and 100 nM), scrambled peptide (100 nM), and rh-bFGF (100 ng/mL) solutions. Each wound was treated with vehicle (20 μL, PBS), rh-bFGF (20 μL), different concentrations of AW1 (20 μL), or scrambled peptide (20 μL) twice a day from days 0–8, respectively. **B–E** Skin wound repair rate on days 0, 2, 4, 6, and 8. Data are expressed as mean ± standard error of the mean (SEM) from six mice (*n* = 6). **F** Expression of Ki67 in neoepidermis after PBS, rh-bFGF, AW1 (1, 10, and 100 nM), and scrambled peptide treatment on day 8 postoperation. Red arrows indicate positive staining, scale bar 50 μm. **G** Quantitative expression of Ki67 in mouse skin wounds on day 8. Data are expressed as mean ± standard error of the mean (SEM) from six mice (*n* = 6). **H** Representative images of keratinocyte scratch wound healing under PBS, rh-bFGF (100 ng/ mL), AW1 (1, 10, and 100 nM), and scrambled peptide (100 nM) treatment at 0 h and 24 h, scale bar 10 μm. **I** Quantification of keratinocyte scratch repair rate under AW1 (1, 10, and 100 nM) treatment for 24 h. Data are expressed as mean ± standard error of the mean (SEM) from three independent experiments (*n* = 3). Ns, no significance; *P < 0.05, **P < 0.01, ***P < 0.001, and ****P < 0.0001 indicate statistically significant difference compared with vehicle

**Correct Figure S5**:

**Fig. S5:**
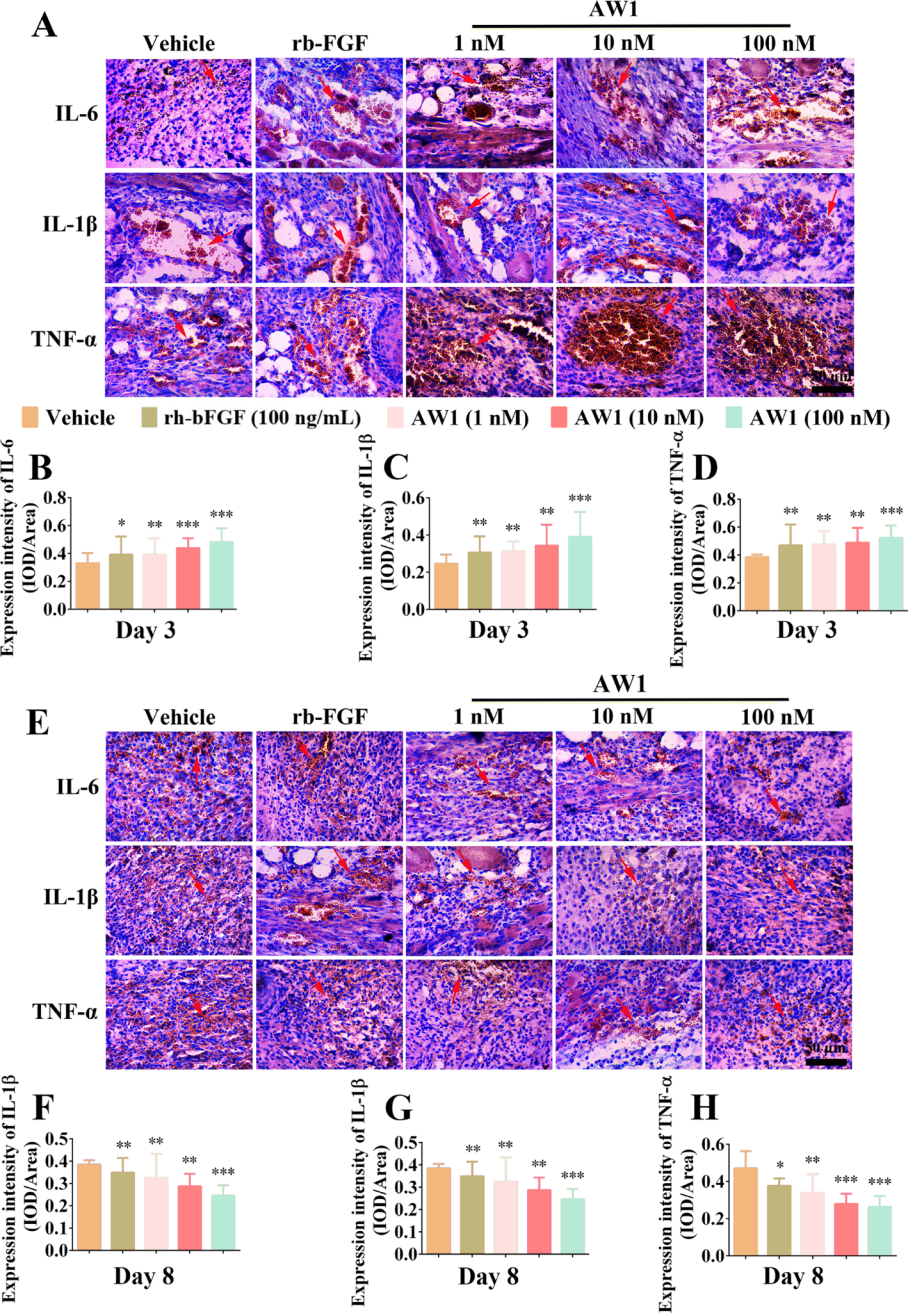
AW1 regulated inflammatory response intensity changes in deep-second degree burns

**Correct Figure S6**:

**Fig. S6:**
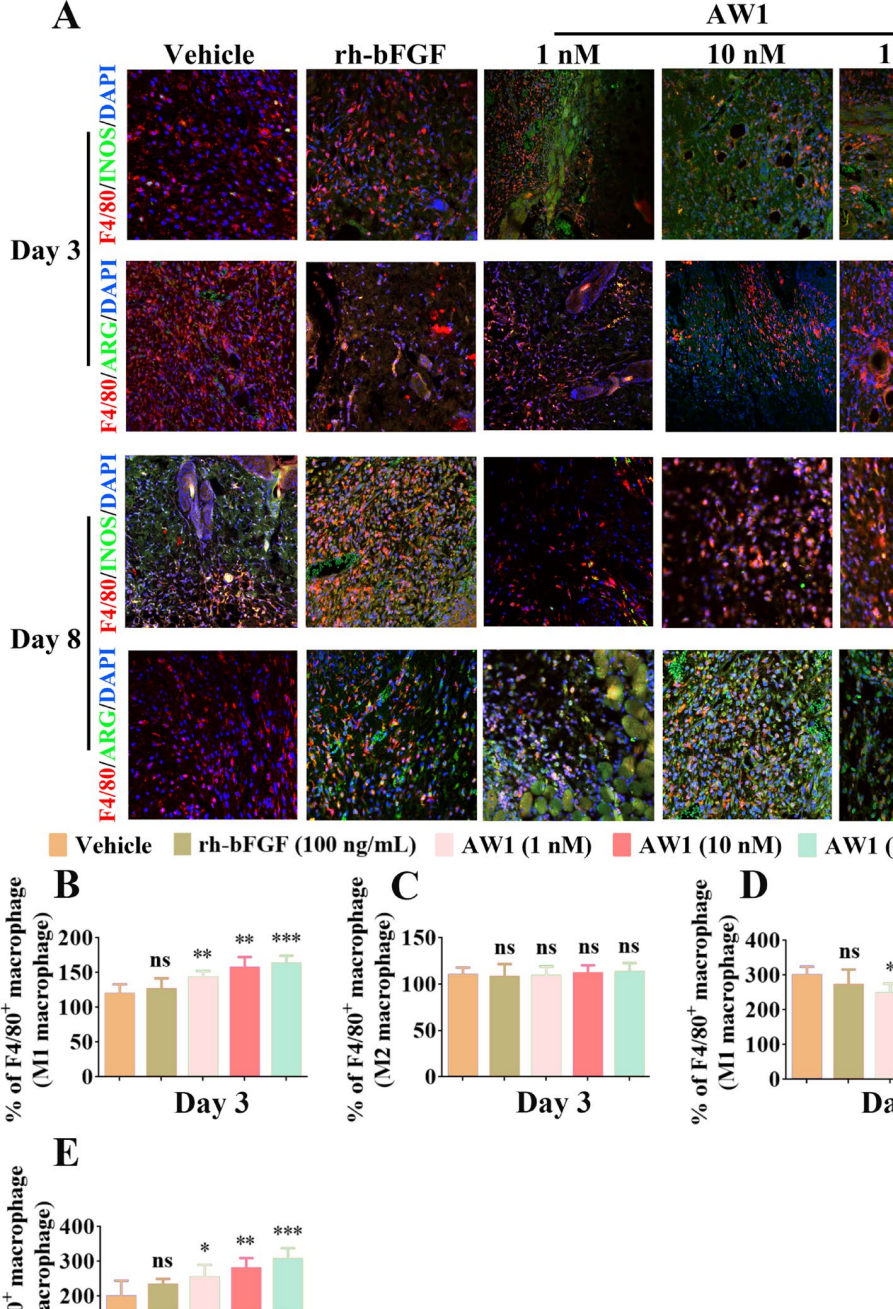
AW1 regulated macrophage polarization and promoted macrophage transition from MI to MII phenotype
