# Supplementary material for: The direct binding of bioactive peptide Andersonin-W1 to TLR4 expedites the healing of diabetic skin wounds
Source: Cell Mol Biol Lett. 2024 Feb 5;29:24. doi: 10.1186/s11658-024-00542-4 (PMC10845795; doi:10.1186/s11658-024-00542-4)
Supplement: Supplementary file 1 — Additional file 1: Figure S1. Chemical structure formula and the advanced structure of AW1. Figure S2. AW1 promoted reepithelialization to accelerate full-thickness wound repair. Figure S3. AW1 inhibited excessive inflammation in full-thickness wound. Figure S4. AW1 promoted proliferation and migration of keratinocyte, macrophage proliferation, and tube formation of HUVEC. Figure S5. AW1 regulated inflammatory response intensity changes in deep-second degree burns. Figure S6. AW1 regulated macrophage polarization and promoted macrophage transition from MI to MII phenotype. Figure S7. Representative images of molecular docking between TLR4 and AW1. Figure S8. AW1 inhibited excessive inflammation in diabetic skin wound healing. Table S1. TLR4-AW1 binding free energy. Table S2. Fasting blood glucose of modeled diabetic mice on different days. [file 11658_2024_542_MOESM1_ESM.docx]

**Additional file 1**

**
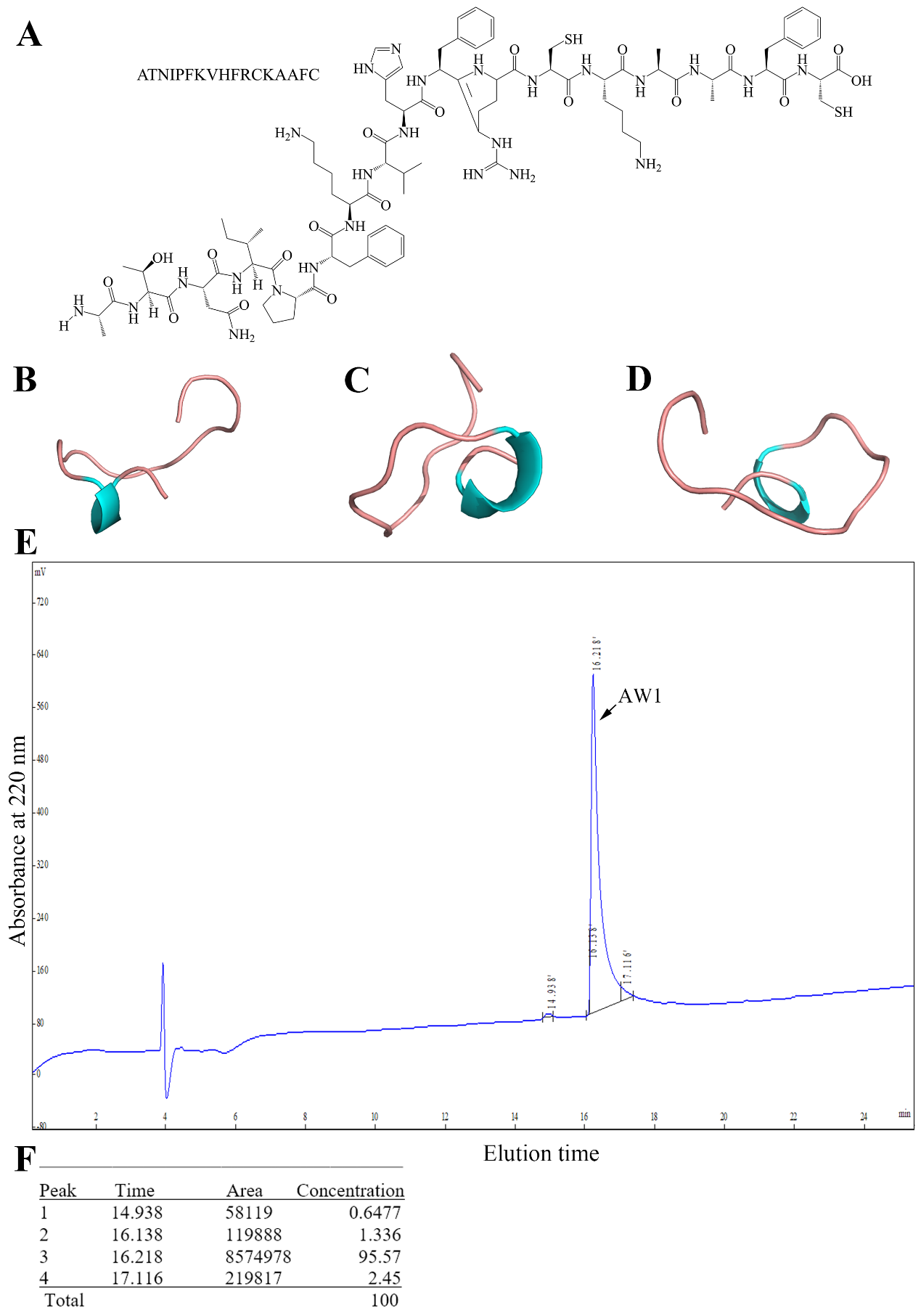
**

Figure S1. Chemical structure formula and the advanced structure of AW1.

A. Chemical structure formula of AW1.

B-D. Different views of advanced structure of AW1 predicted by PEP-FOLD3.

E-F. The purity of AW1 was test by RP-HPLC. The AW1 wad applied to a C18 RP-HPLC column pre-equilibrated with 0.1% (v/v) trifluoroacetic acid (TFA) in water, the elution was achieved by a linear gradient of 0.1% (v/v) TFA in acetonitrile at a flow rate of 1.0 mL/min and monitored at 220 nm. AW1 was monitored in the peak 3 at 16.218 min. Quantification the ranks in the HPLC, and the purity of AW1 was 95.57%.


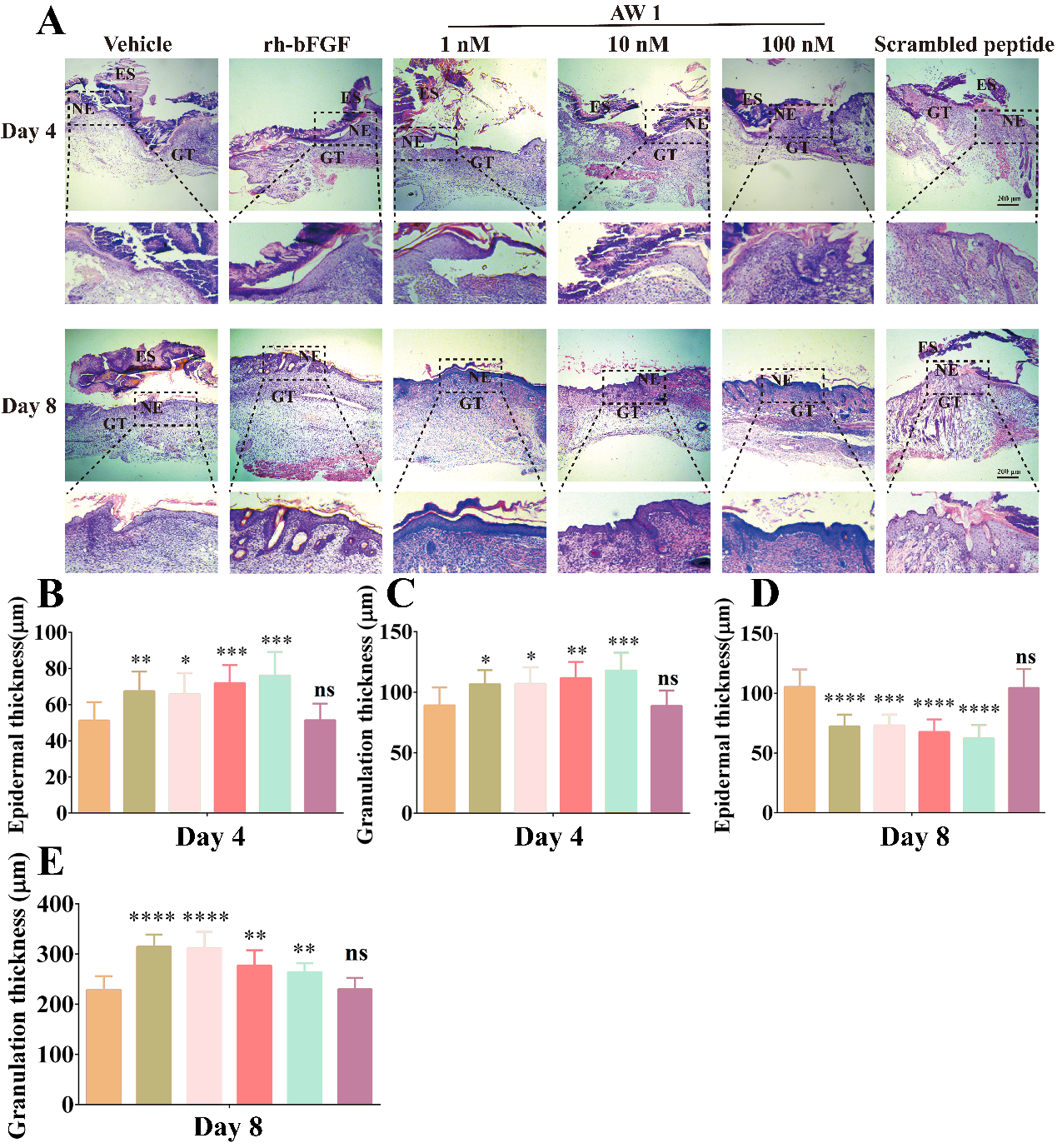


Figure S2. AW1 promoted re-epithelialization to accelerate full-thickness wound repair.

A. Representative figure of H&E staining of full-thickness wound on days 4 and 8. Es: eschar; NE: neoepidermis; GT: regenerated granulation tissue; scale bar 20 μm.

B-E. Quantification of neoepidermis and new granulation tissue thickness of full-thickness wound on days 4 and 8.

All data are expressed as mean ± standard error of the mean (SEM) from 6 mice (n = 6); ns, no significance, **P <* 0.05, ***P <* 0.01, ****P <* 0.001, and *****P <* 0.0001 indicate statistically significant difference compared to vehicle.

**
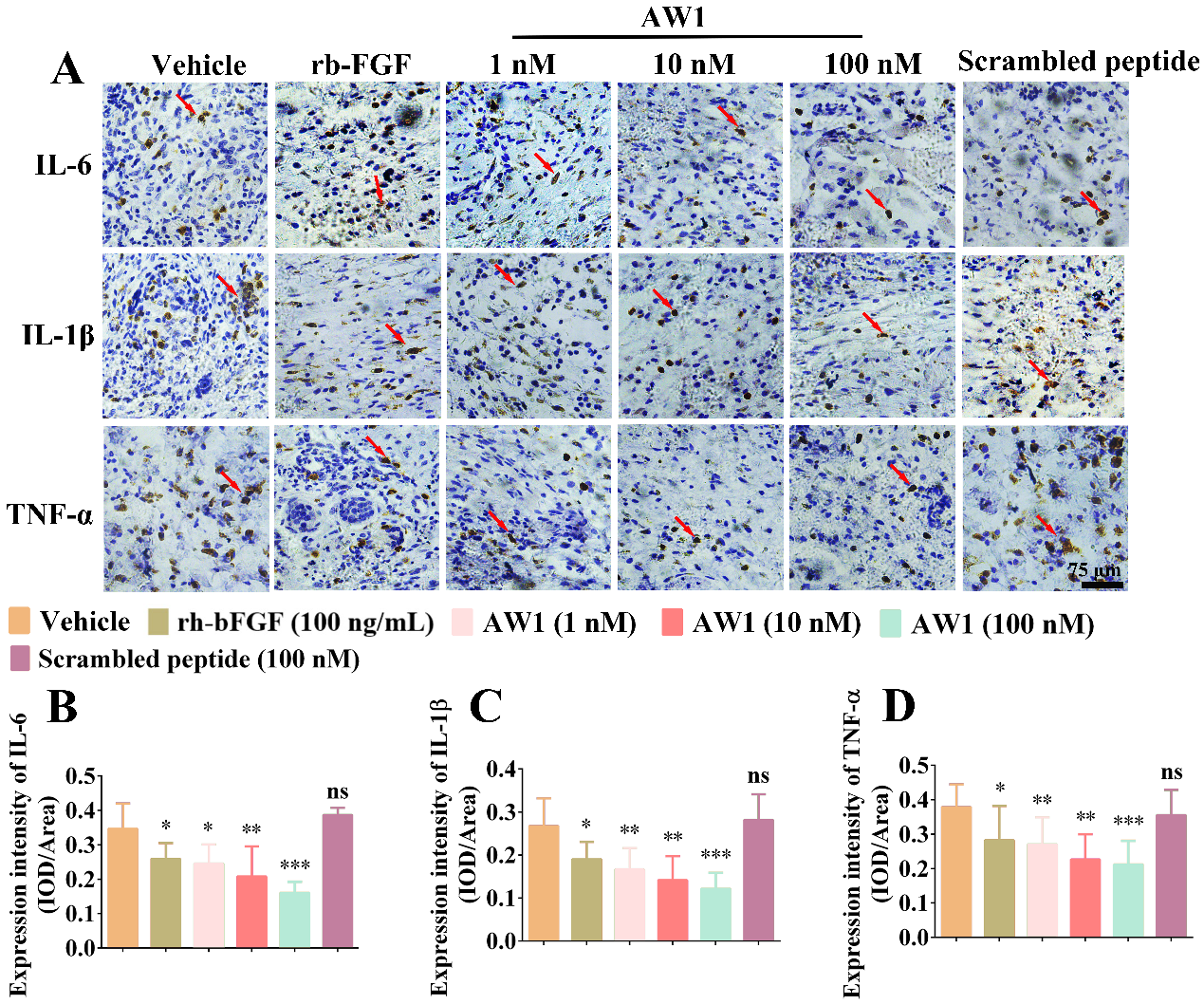
**

Figure S3. AW1 inhibited excessive inflammation in full-thickness wound.

A. Immunohistochemical analysis of IL-6, IL-1β, and TNF-α expression in skin wounds on day 8. Red arrows indicate positive staining, scale bar 75 μm.

B-D. Quantitative expression of IL-6, IL-1β, and TNF-α in mouse skin wounds on day 8.

All data are expressed as mean ± SEM from 6 mice (n = 6); ns, no significance, **P <* 0.05, ***P <* 0.01, and ****P <* 0.001 indicate statistically significant difference compared to vehicle.

**
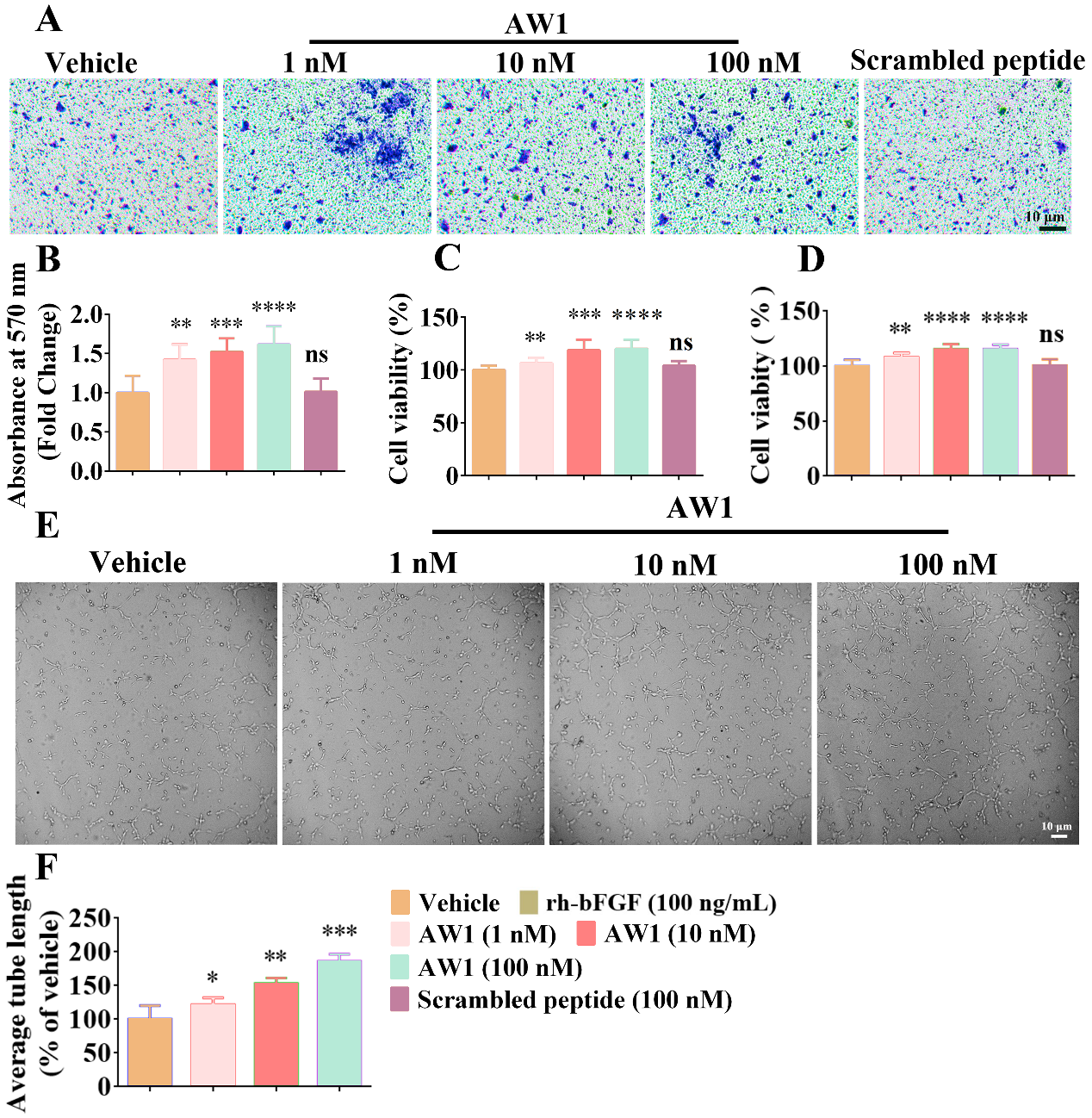
**

Figure S4. AW1 promoted proliferation and migration of keratinocyte, macrophage proliferation, and tube formation of HUVEC.

A. Representative images showing the promoting keratinocyte migration ability of AW1 (1, 10, and 100 nM). Scale bar = 10 μm.

B. Quantification of pro-migratory effects of AW1 (1, 10, and 100 nM) against keratinocytes. The migrated keratinocyte was stained 0.1% crystal violet, eluted by 33% glacial acetic acid. Finally, the absorbance was detected at 570 nm to indirectly reflect the number of migrated cells. The data represent as the relative absorbance of the vehicle at 570 nm.

C. Quantitative pro-proliferative effects of AW1 (1, 10, and 100 nM) on keratinocytes. The data represent as the relative cell viability of the vehicle.

D. Quantitative the proliferation-promoting effects of AW1 (1, 10, and 100 nM) on macrophages. The data represent as the relative cell viability of the vehicle.

E. Representative images of HUVECs tube formation. Scale bar = 10 μm.

F. Quantitative analysis of the tube formation assay. The values of the average tube length were measured and the data represent as the relative length of the vehicle.

All data are expressed as mean ± SEM from three independent experiments (n=3); ns, no significance, **P <* 0.05, ***P <* 0.01, and ****P <* 0.001 indicate statistically significant difference compared to vehicle.


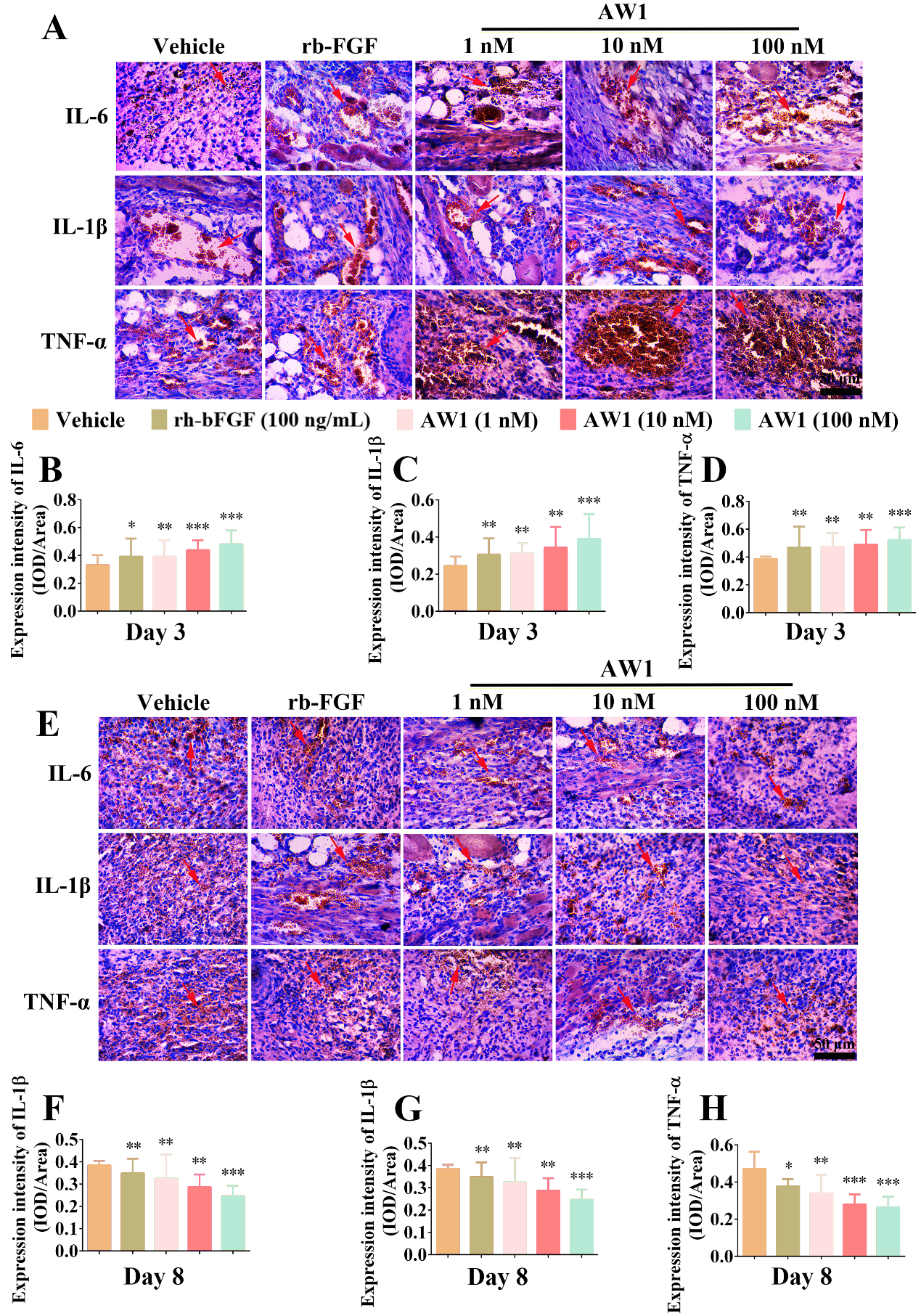


Figure S5. AW1 regulated inflammatory response intensity changes in deep-second degree burns.

A. Immunohistochemical images of IL-6, IL-1β, and TNF-α-positive staining of deep second-degree burn on day 3. Red arrows indicate positive staining, scale bar 50 μm.

B-D. Quantitative expression of IL-6, IL-1β, and TNF-α in mouse skin wounds on day 3.

E. Immunohistochemical images of IL-6, IL-1β, and TNF-α-positive staining of deep second-degree burn on day 8. Red arrows indicate positive staining, scale bar 50 μm.

F-H. Quantitative expression of IL-6, IL-1β, and TNF-α in mouse skin wounds on day 8.

All data are expressed as mean ± SEM from 6 mice (n = 6); **P* < 0.05, ***P* < 0.01, and ****P* < 0.001 indicate statistically significant difference compared to vehicle.


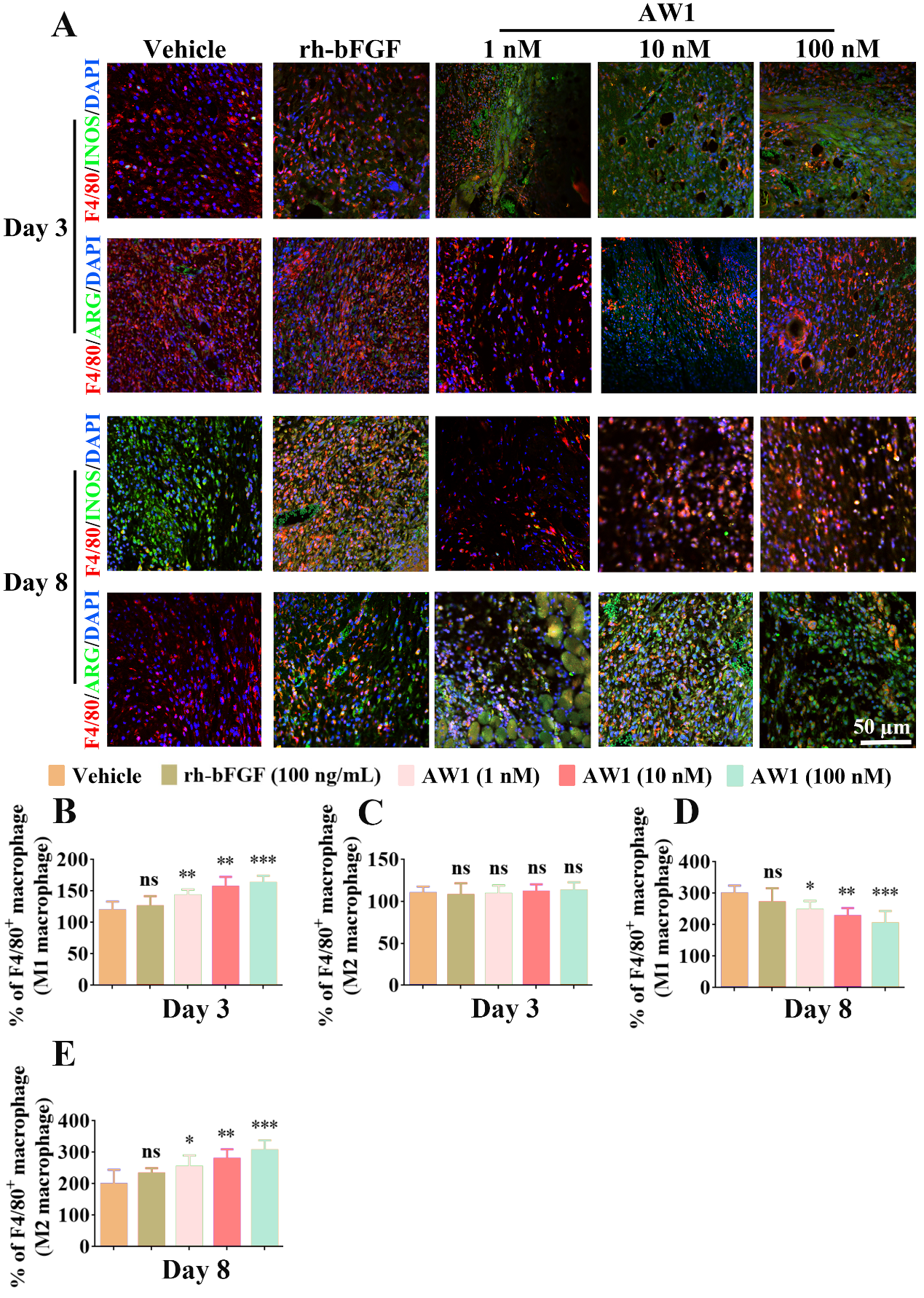


Figure S6. AW1 regulated macrophages polarization and promoted macrophage transition from MⅠ to MⅡ phenotype.

A. Representative immunofluorescence images of macrophages (F4/80), MⅠ macrophages (F4/80/iNOS), and MⅡ macrophages (F4/80/Arg) in wound tissues under PBS, rh-bFGF (100 ng/mL), or AW1 (1, 10, and 100 nM) treatment on days 3 and 8. F4/80 red fluorescence for macrophages, INOS green fluorescence for MⅠ macrophages, ARG green fluorescence for MⅡ macrophages, and DAPI blue fluorescence for nuclei; scale bar 50 µm.

B-C. Quantification of positive-staining intensity of MⅠ and MⅡ phenotype macrophages on day 3.

D-E. Quantification of positive-staining intensity of MⅠ and MⅡ phenotype macrophages on day 8.

All data are expressed as mean ± SEM from 6 mice (n = 6), ns, no significance, **P* < 0.05, ***P* < 0.01, and ****P* < 0.001 indicate statistically significant difference compared to vehicle.


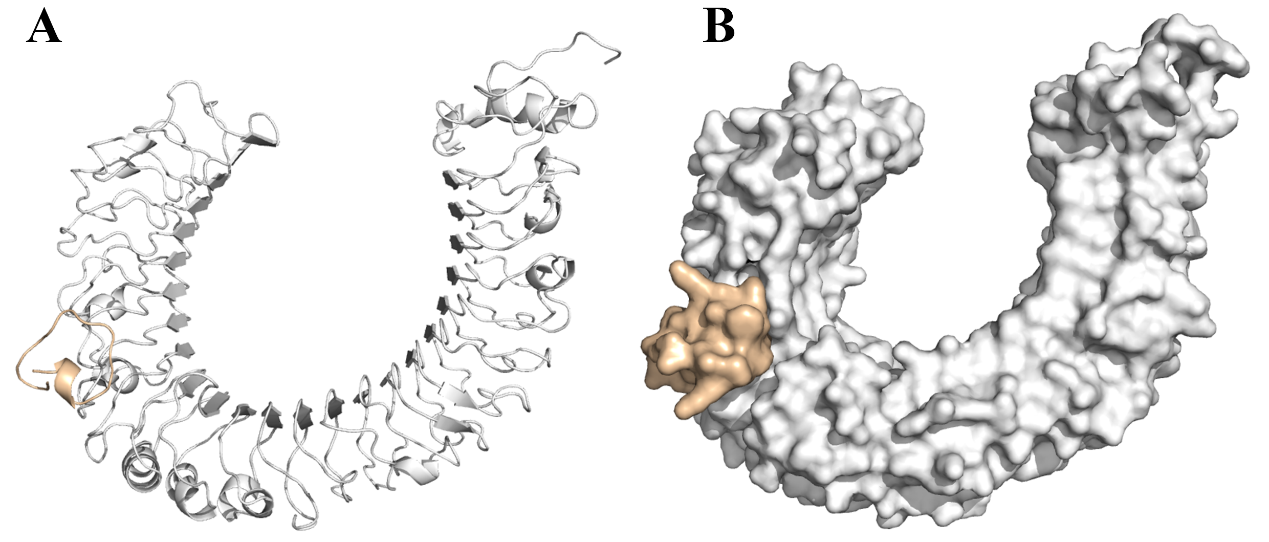


Figure S7. Representative images of molecular docking between TLR4 and AW1.

A-B. Representative images of molecular docking binding sites between TLR4 and AW1. White represents TLR4 and gold represents AW1.


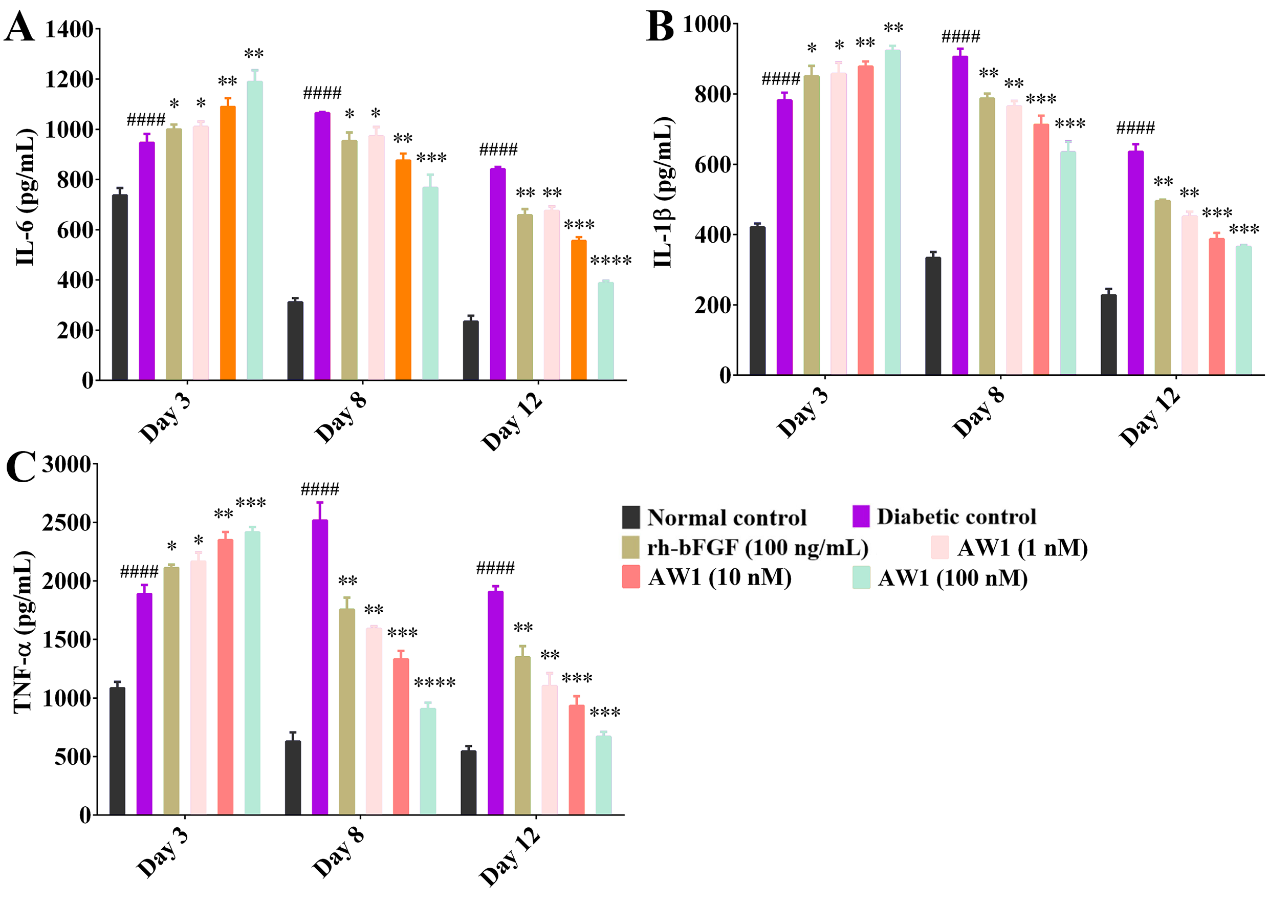


Figure S8. AW1 inhibited excessive inflammation in diabetic skin wound healing.

A. Effect of AW1 on IL-6 expression on days 3, 8, and 12.

B. Effect of AW1 on IL-1β expression on days 3, 8, and 12.

C. Effect of AW1 on TNF-α expression on days 3, 8, and 12.

All data are expressed as mean ± SEM from 6 mice (n = 6); ^####^*P <* 0.0001 indicates statistically significant difference compared to normal control; **P <* 0.05, ***P <* 0.01, ****P <* 0.001, and *****P <* 0.0001 indicate statistically significant difference compared to diabetic control.

Table S1. TLR4-AW1 binding free energy

| **Location** | **van der Waals** | **Electrostatic** | **Polar Solvation** | **Non-Polar Solvation** | **TOTAL** |
| --- | --- | --- | --- | --- | --- |
| R PRO27 | 0 | 12.133 | -12.12503846 | 0 | 0.007961538 |
| R CYX28 | -7.69E-05 | 0.175884615 | -0.175307692 | 0 | 0.0005 |
| R ILE29 | -0.000115385 | -0.133423077 | 0.133 | 0 | -0.000538462 |
| R GLU30 | -7.69E-05 | -11.09923077 | 11.09403846 | 0 | -0.005269231 |
| R VAL31 | -0.000269231 | 0.184076923 | -0.183423077 | 0 | 0.000384615 |
| R VAL32 | -3.85E-05 | -0.125076923 | 0.124923077 | 0 | -0.000192308 |
| R PRO33 | -3.85E-05 | -0.035230769 | 0.035230769 | 0 | -3.85E-05 |
| R ASN34 | 0 | -0.021307692 | 0.021423077 | 0 | 0.000115385 |
| R ILE35 | -7.69E-05 | -0.136576923 | 0.136346154 | 0 | -0.000307692 |
| R THR36 | -0.000153846 | 0.155423077 | -0.154769231 | 0 | 0.0005 |
| R TYR37 | -0.000115385 | -0.1625 | 0.162153846 | 0 | -0.000461538 |
| R GLN38 | -0.0005 | 0.3305 | -0.328846154 | 0 | 0.001153846 |
| R CYX39 | -0.000269231 | -0.014961538 | 0.014884615 | 0 | -0.000346154 |
| R MET40 | -0.000576923 | 0.3065 | -0.304846154 | 0 | 0.001076923 |

Table S2. Fasting blood glucose of modeled diabetic mice on different days

| **Days** | **Fasting blood glucose (mmol/L)** |
| --- | --- |
| 3 | 13.5, 10.6, 9.1, 9.4, 11.0, 11.4, 9.7, 9.2, 8.4, 10.1, 11.9, 10.4, 8.5, 11.4, 11.3, 12.2, 11.9, 8.7, 8.9, 11.2, 11.9, 9.9, 13.8, 9.3, 10.2, 11.6, 9.5, 8.9, 10.3, 9.4 |
| 7 | 13.0, 9.2, 9.8, 11.2, 9.9, 12.7, 11.8, 11.2, 12.7, 17.1, 11.7, 12.8, 10.9, 9.8, 11.5,  12.3, 10.4, 10.7, 10.9, 11.4, 10.4, 11.8, 12.3, 15.1, 15.9, 11.5, 10.9, 11.9, 13.5 |
| 14 | 15.1, 11.9, 13.7, 12.8, 12.7, 14.0, 14.9, 13.7, 17.1, 13.8, 13.5, 15.2, 12.7, 13.8  15.3, 15.9, 16.8, 14.1, 13.8, 15.3, 14.9, 16.8, 17.3, 16.9, 15.5, 16.0, 14.6, 13.9 |
| 22 | 14.8, 18.1, 17.3 18.2, 20.2, 15.0, 15.9, 16.7, 15.8, 16.1, 17.5, 16.6, 14.9, 16.9,  15.7, 16.2, 16.8, 14.9, 18.9, 16.0, 17.3, 16.7, 17.4, 20.6, 23.7, 19.8, 17.4, 16.9 |
| 30 | 17, 20, 18.2, 19.1, 17.7, 17.3, 22.6, 17.3, 19.8, 16.9, 17.9, 25.1, 22.3, 18.2, 17.4, 17.5, 19.2, 21.7, 20.9, 17.1, 17.4, 17.8, 19.2, 19.8, 17.6, 18.9, 19.7, 17.5,  17.3, 18.9, 17.7, 18.2, 19.9, 23.8, 24.6, 25.9, 22.5, 21.6, 25.3, 20.8, 23.4, 19.7,  25.3, 24.2, 26.5,17.9, 19.5, 19.7, 19.6, 24.5, 20.6, 17.8, 18.5, 27.3, 28.4, 25.7  24.5, 22.7, 19.6, 18.4, 19.3 |

After one month feeding with a high-fat diet, C57BL/6 mice were intraperitoneally injected with streptozotocin (STZ, Solarbio, China, 30 mg/kg/day) for five consecutive days. Blood glucose was then monitored at days 3, 7, 14, 22, and 30 to ensure successful construction of type 2 diabetic mice.
